# Thermosensitive pilus production by FCT type 3 *Streptococcus pyogenes* controlled by Nra regulator translational efficiency

**DOI:** 10.1111/mmi.14408

**Published:** 2019-11-11

**Authors:** Masanobu Nakata, Tomoko Sumitomo, Nadja Patenge, Bernd Kreikemeyer, Shigetada Kawabata

**Affiliations:** ^1^ Department of Oral and Molecular Microbiology Osaka University Graduate School of Dentistry 1‐8, Yamadaoka Suita Osaka 565‐0871 Japan; ^2^ Institute of Medical Microbiology, Virology and Hygiene University of Rostock Rostock D‐18057 Germany

## Abstract

*Streptococcus pyogenes* produces a diverse variety of pili in a serotype‐dependent manner and thermosensitive expression of pilus biogenesis genes was previously observed in a serotype M49 strain. However, the precise mechanism and biological significance remain unclear. Herein, the pilus expression analysis revealed the thermosensitive pilus production only in strains possessing the transcriptional regulator Nra. Experimental data obtained for *nra* deletion and conditional *nra*‐expressing strains in the background of an M49 strain and the *Lactococcus* heterologous expression system, indicated that Nra is a positive regulator of pilus genes and also highlighted the importance of the level of intracellular Nra for the thermoregulation of pilus expression. While the *nra* mRNA level was not significantly influenced by a temperature shift, the Nra protein level was concomitantly increased when the culture temperature was decreased. Intriguingly, a putative stem‐loop structure within the coding region of *nra* mRNA was a factor related to the post‐transcriptional efficiency of *nra* mRNA translation. Either deletion of the stem‐loop structure or introduction of silent chromosomal mutations designed to melt the structure attenuated Nra levels, resulting in decreased pilus production. Consequently, the temperature‐dependent translational efficacy of *nra* mRNA influenced pilus thermoregulation, thereby potentially contributing to the fitness of *nra*‐positive *S. pyogenes* in human tissues.

## Graphical abstract

Thermosensitive pilus production from a distinct *Streptococcus pyogenes* subset is reliant on the post‐transcriptional regulation of the positive regulator Nra, where a putative stem‐loop structure within the coding region of *nra* mRNA functions as a thermosensor to modulate the translational efficiency of *nra* mRNA via potential interactions with the translation initiation complex. This type of regulation highlights the underlying mechanism used by the pathogen to establish infection and enhance fitness in host tissues.

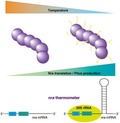

## Introduction

Microorganisms possess intricate mechanisms to expeditiously respond to changes in environmental conditions, such as temperature, pH, salinity, pressure, nutrition and oxygen availability, thus allowing them to adapt to environmental shifts and avoid deleterious consequences (Bleuven and Landry, 2016; Fang *et al.*, 2016; Papadimitriou *et al.*, 2016; Kraemer and Boynton, 2017). During the course of an infection, human pathogenic bacteria successfully adapt to anatomical niches and exhibit virulence through the modulation of virulence factor gene expression (Richardson *et al.*, 2015; Rossi *et al.*, 2018). When bacteria are systemically disseminated from the host external surface, their surrounding temperature is significantly increased inside host tissues. Such temperature shifts enable bacteria to frequently modulate transcription, translation and DNA replication efficiency, thus contributing to pathological processes (de Mendoza, 2014; Guijarro *et al.*, 2015).

*Streptococcus pyogenes*, a Lancefield group A *Streptococcus* organism, is a mesophilic human pathogen with diverse clinical manifestations, including a broad spectrum of infections ranging from uncomplicated self‐limiting purulent diseases, such as pharyngitis and pyoderma, to more life‐threatening invasive and autoimmune diseases (Cunningham, 2000). Historically, two major schemes have been utilized to classify *S. pyogenes* serotypes. One is based on the antigenicity of the M protein, a major virulence‐associated surface protein with a variable region in the N‐terminus (Fischetti *et al.*, 1988). Based on the DNA sequence diversity of the 5′ variable region of the *emm* gene encoding M protein, *emm* types are currently classified into over 220 types (Beall *et al.*, 1996; Sanderson‐Smith *et al.*, 2014). The other typing scheme, T serotyping, is based on the antigenicity of T antigens, that is, trypsin‐resistant antigens (Griffith, 1934). Pilus subunits have been shown to confer antigenicity during T serotyping, with the major subunit reported to be a major serotype determinant (Falugi *et al.*, 2008). Thus, the variability of *S. pyogenes* pili and their relationship with disease diversity, as well as human tissue tropism of *S. pyogenes* have been examined (Bessen, 2016).

Since their discovery, *S. pyogenes* pili have been shown to promote bacterial adhesion to both human tonsil tissues and human keratinocytes, while they are also known to participate in the biofilm formation and evasion of the host immune system in a serotype‐specific manner (Mora *et al.*, 2005; Abbot *et al.*, 2007; Nakata *et al.*, 2009; Kimura *et al.*, 2012; Rouchon *et al.*, 2017; Tsai *et al.*, 2017). These multiple roles indicate a crucial function of pili during the onset of *S. pyogenes* infection. Pilus biogenesis genes are located in a specific genomic region known as the FCT region, which encodes fibronectin‐binding proteins, collagen‐binding proteins and T antigens (Bessen and Kalia, 2002). Based on the heterogeneity of the genetic composition and sequences, this region is currently classified into nine types, with strains belonging to the same *emm* type basically sharing the same types of FCT regions (Kratovac *et al.*, 2007). In this region, three different transcriptional regulators are encoded, that is, Nra, RofA and MsmR (Fogg *et al.*, 1994; Podbielski *et al.*, 1999; Nakata *et al.*, 2005). The Nra transcriptional regulator is specific for FCT types 3 and 8, while the gene encoding RofA, whose amino acids exhibit a 62% identity with Nra, is present in all of the other FCT types. The third regulator, MsmR, is specific for FCT types 3, 4, 7 and 8. Although previous reports have indicated that the expression of pilus genes is regulated by Nra in FCT type 3 strains (Podbielski *et al.*, 1999; Luo *et al.*, 2008), the mechanism of regulation remains controversial. Luo, *et al*., reported a positive regulator function for Nra in an M53 strain, whereas in a serotype M49 strain it was shown to be a negative regulator of the gene encoding the minor pilus subunit Cpa (Podbielski *et al.*, 1999; Luo *et al.*, 2008). Previously, we reported the induction of pilus gene expression at low temperatures in a serotype M49 strain. However, whether this thermosensitive phenotype is possessed by other serotype strains remains unknown and the underlying mechanism of the phenotype also remains elusive.

In contrast to the previous report, the present investigation showed that in low culture temperatures, Nra functions as a positive regulator of pilus biogenesis genes in the background of a serotype M49 strain. Among the various tested strains, thermosensitive pilus production was only observed in FCT‐3 type Nra‐positive strains. It is also interesting to note that a putative stem‐loop structure within the coding region of *nra* mRNA was shown to be involved in the temperature‐dependent translation of *nra* mRNA. The biological significance of thermosensitive pilus expression was also highlighted by findings showing that the pilus production increased the rates of adherence to human keratinocytes and survival in human blood. Thus, we propose that the Nra‐dependent regulation of thermosensitive pilus production supports the fitness of a specific subset of *S. pyogenes* in the host.

## Results

### FCT type‐dependent thermosensitive pilus production

**Figure 1:**
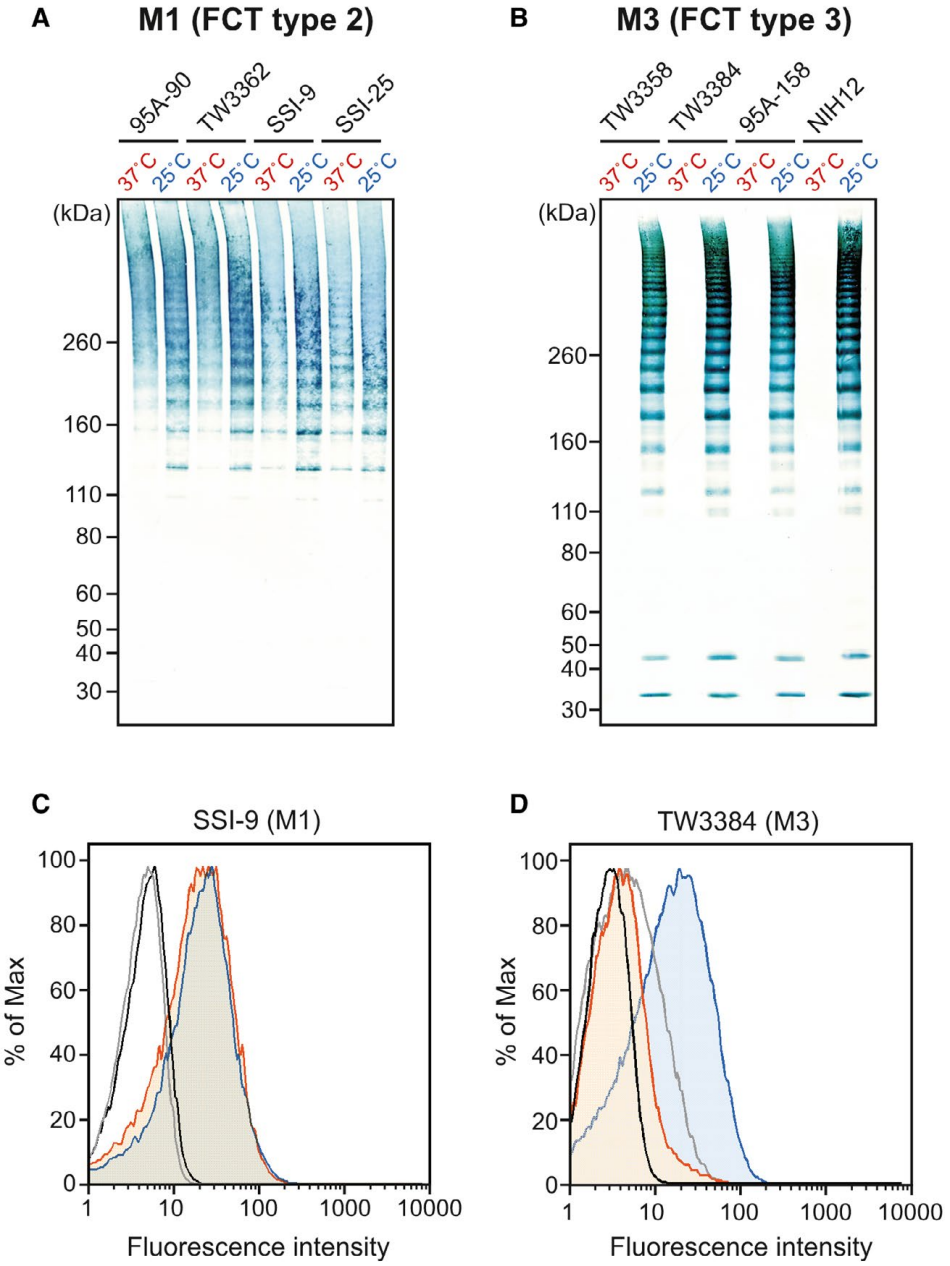
Influence of culture temperature on the pilus production by serotype M1 and M3 strains. (A, B) Pilus production of *S. pyogenes* serotype M1 and M3 strains grown at 37°C or 25°C was examined by the immunoblot analysis. Cell wall fractions of four clinical isolates were extracted with mutanolysin and utilized for the immunoblot analysis with anti‐FctA antiserum. Protein marker sizes are indicated on the left. (C, D) The surface display of FctA was examined using Fluorescence‐activated cell sorter analysis with cells grown to the exponential phase grown at 37°C or 25°C. FctA was labeled with mouse anti‐FctA serum and FITC‐conjugated goat anti‐mouse IgG. Orange and blue histograms represent data for cells cultured at 37°C and 25°C respectively. Results obtained with nonimmune serum served as a negative control (black line, 37°C; gray line, 25°C).

We previously reported that a serotype M49 strain categorized as FCT type 3 efficiently produced detectable pili at temperatures below 30°C, but not at 37°C (Nakata *et al.*, 2009). To determine whether other serotypes exhibit such thermosensitivity, the clinically relevant M1 (FCT type 2) and M3 (FCT type 3) strains were cultured at both 37 and 25°C, then the pilus production was examined by immunoblot and flow cytometry analyses using antiserum against the corresponding major pilus subunit, FctA (Fig. 1). While all tested M1 strains produced pili at both temperatures (Fig. 1A and C), the M3 strains exhibited no detectable pili after culturing at 37°C (Fig. 1B and D). Moreover, when the culture temperature was decreased, detectable T3 production was induced, similar to the expression pattern of T49 pili (Fig. 1B). Immunoblot analysis was also conducted for other serotypes, which are listed in Supplemental Table S1. All tested FCT types 1, 4, 5, 6 and 7 strains produced pili, regardless of the culture temperature (Table S1). In contrast, the temperature‐dependent pilus detection was observed with the FCT type 3 strains (M5, M49, M52, M67 and M71) (Table S1). Thus, all of the tested FCT type 3 strains, except for the M18 strains, produced pili (Table S1), which led us to form a hypothesis stating that unknown factors specific for FCT type 3 strains are responsible for the temperature‐sensitive pilus production.

### Thermoregulated pilus production by M49 strain promotes adherence to keratinocytes and survival in human blood

**Figure 2:**
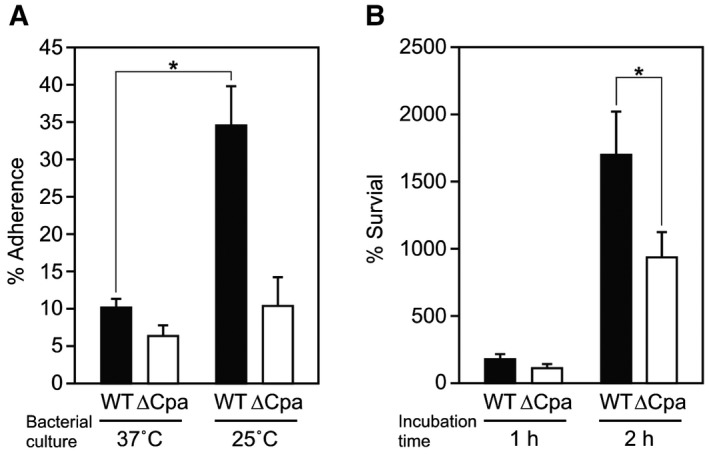
Effects of culture temperature on pilus‐dependent adherence to human keratinocytes and bacterial survival rate in human blood of serotype M49 strain. A. M49 strain 591 (WT) and its isogenic *cpa* operon deletion mutant strain (∆Cpa) were grown to the exponential phase at 37°C or 25°C. HaCaT cells were infected with bacteria at an MOI of 10 at 37°C for 2 h. Infected cells were lysed and cell‐associated bacteria were recovered to determine bacterial adherence rates, shown as a percent of inoculum CFUs. Representative results are shown from three independent experiments performed with triplicate samples, with values presented as the mean ± SD. *P < 0.01. B. Human heparinized blood was combined with 200 CFUs of WT or ∆Cpa grown to the exponential phase at 25°C, then incubated for 2 h. Survival rates are presented as a percent of inoculum CFUs. Representative results are shown from three independent experiments performed with triplicate samples, with values presented as the mean ± SD. *P < 0.01.

When bacteria systemically disseminate from the initial infection site, that is, the upper respiratory tract and skin, the temperature in the vicinity of the bacteria increases and thus pilus production by FCT type 3 strains is concomitantly halted. Pilus production would be advantageous for FCT type 3 strains when the temperature at the initial infection site is lower, while it would be detrimental at the dissemination stage when the temperature increases. To obtain experimental evidence in support of our hypothesis, we initially examined the ability of a serotype M49 wild‐type (WT) strain and its isogenic deletion mutant strain of pilus gene operon (∆Cpa) to adhere to human keratinocyte HaCaT cells (Fig. 2A). As compared to data obtained with the WT strain cultured at 37°C, adherence when cultured at 25°C was remarkably increased. In contrast, the rates of adherence of ∆Cpa were not statistically different between the different temperatures. These results partially support the notion that pilus‐dependent adherence of the tested M49 strain to human keratinocytes occurs only in low‐temperature conditions. Next, a blood survival assay was performed using human blood and the same set of bacteria was cultured at 25°C (Fig. 2B). Unexpectedly, the survival rate of the ∆Cpa strain was lower than that of the WT strain after 2 h. Thus, though these findings do not clearly prove our hypothesis, they suggest that T49 pili promote bacterial adherence to human tissues, as well as survival and proliferation in human blood.

### Deletion of *nra* abolishes T49 pilus expression at low temperatures and culture temperature has effect on the Nra protein level

We previously reported that the temperature‐dependent pilus production by a serotype M49 strain was transcriptionally regulated, while other reports have shown involvement of the Nra transcriptional regulator in the pilus gene regulation (Podbielski *et al.*, 1999; Luo *et al.*, 2008). Of the nine FCT type strains, *nra* distribution is restricted to types 3 and 8. In addition, a point‐nonsense mutation in the Nra W218‐coding codon of M3 and M49 strains has been reported in a serotype M18 strain (Nakata *et al.*, 2005; Calfee *et al.*, 2018). Therefore, we postulated that Nra is involved in the thermoregulation process and performed related examinations.

**Figure 3:**
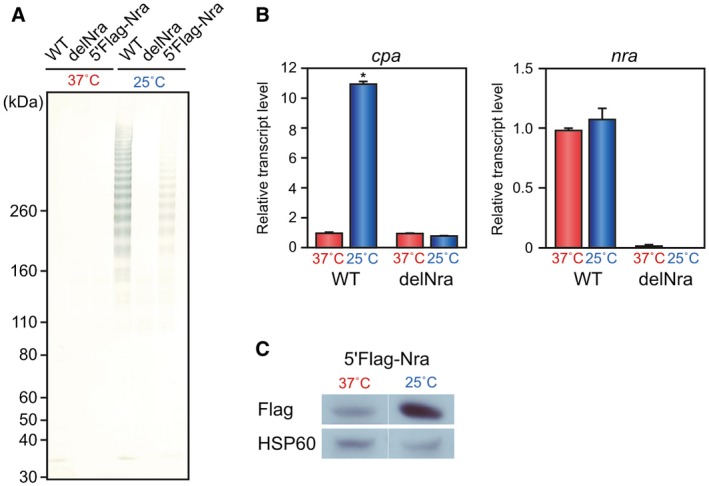
Culture temperature shift induces a change in Nra protein level. A. Cell wall fractions of the M49 strain 591 (WT), an *nra* in‐frame deletion mutant (delNra) and a mutant strain complemented with 5′‐*flag*‐tagged *nra* (5'Flag‐Nra) grown at 37°C or 25°C were immunoblotted with antiserum against FctA. Molecular mass standard sizes are indicated on the left. B. Expression of *cpa* and *nra* in WT and delNra strains grown to the late‐exponential phase at 37°C or 25°C was examined by the real‐time RT‐PCR. Results for *gyrA* served as an internal control. Three independent experiments were performed with triplicate samples and values are presented as the mean ± SD. *P < 0.01. C. Whole‐cell extracts were prepared from 5′Flag‐Nra cultured at 37°C or 25°C, then immunoblotted with anti‐Flag tag mAb. Results obtained with anti‐HSP60 mAb served as a control.

To gain insight into whether Nra is involved in the thermoregulation of pilus production by FCT type 3 strains, an *nra* in‐frame deletion mutant strain (delNra) and a strain chromosomally complemented with 5′‐*flag*‐tagged *nra* (5′Flag‐Nra) were constructed with a WT strain background. Consistent with our previous findings, FctA49 was not clearly detected by the immunoblot analysis of cell wall fractions extracted from any of the strains cultured at 37°C (Fig. 3A). In contrast, notable high molecular weight ladder bands representing FctA49 polymerization were detected in the fractions of the WT strain and, to a lesser extent, in the 5′Flag‐Nra strain when grown at 25°C. However, upon *nra* deletion, the ladder bands completely disappeared, indicating that Nra is responsible for T49 pilus production at lower temperatures. To determine the transcriptional level of the pilus gene operon, the expression level of *cpa*, which encodes a minor pilus subunit, was examined by the RT‐PCR analysis (Fig. 3B). In line with immunoblot results, *cpa* transcript levels for the WT and delNra strains were at the baseline when cultured at 37°C, while increased *cpa* expression was observed in the WT, but not the delNra strain after culturing at 25°C. Therefore, Nra shows transcriptional effects on the expression of pilus operon genes at lower temperatures.

Based on these findings, we considered that either *nra* transcription or protein levels were modulated by the temperature shift. Thus, we initially evaluated *nra* transcription at 37 and 25°C using RT‐PCR analysis, though no differential *nra* expression was observed with either of those culture temperatures (Fig. 3B). Next, the Nra protein level was examined using 5′Flag‐Nra (Fig. 3C). Notably, immunoblot analysis of whole‐cell extracts from each strain after culturing at 37°C or 25°C revealed that the Nra protein level was increased with a decreased culture temperature, whereas the level of Hsp60, a negative control protein, was unchanged by that temperature shift. Thus, it is likely that an increase in the Nra protein level corresponding to a decrease in the temperature is crucial for the thermoregulation of pilus production.

### Intracellular Nra protein level crucial for the pilus production

**Figure 4:**
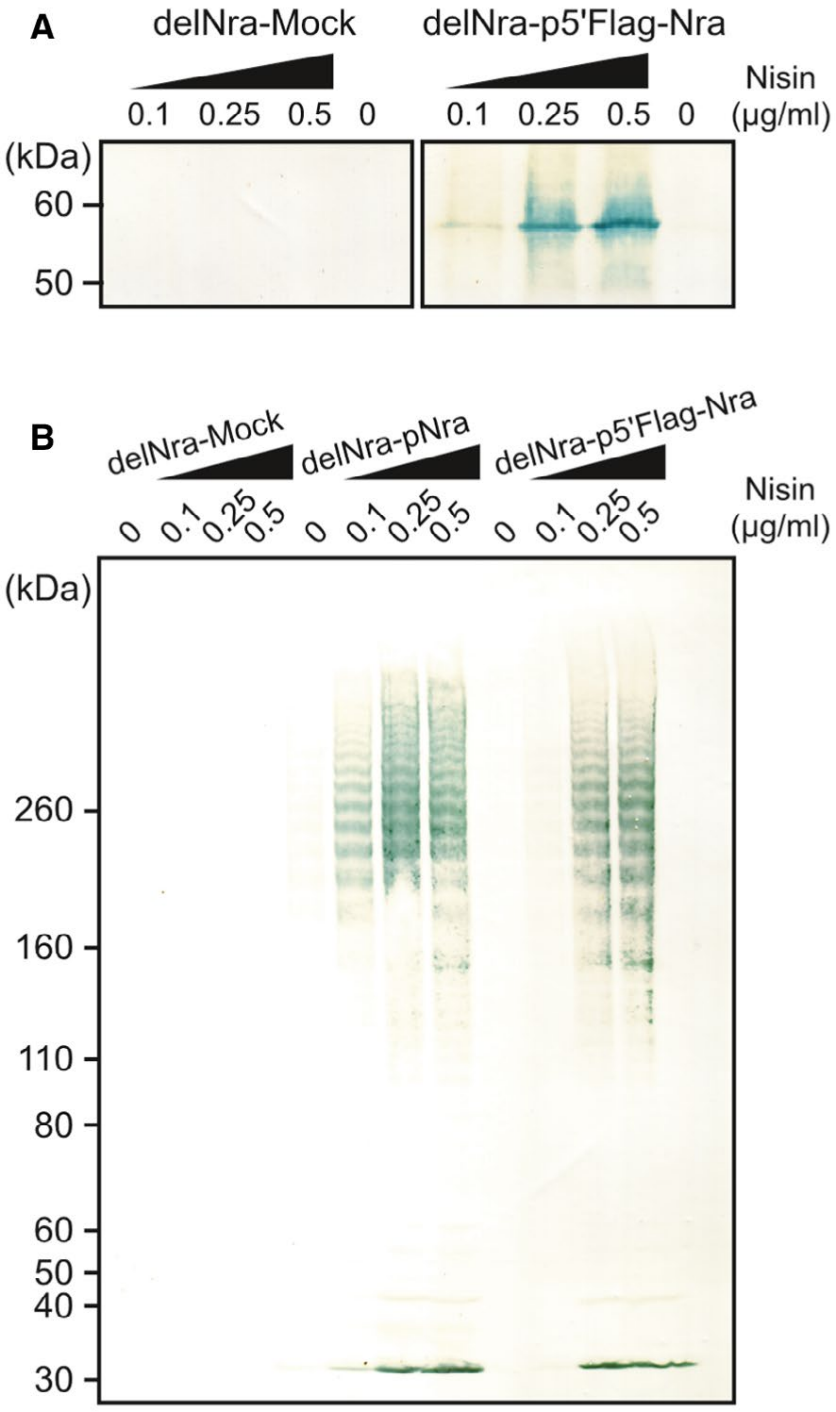
Pilus expression dependent on the intracellular Nra protein level. A. An *nra* deletion mutant strain was transformed with either an empty pMSP3545 shuttle vector (delNra‐Mock) or pMSP3545‐Flag‐*nra* (delNra‐p5′Flag‐Nra), then grown at 37°C to the exponential phase. Nisin was added at the indicated concentrations, then incubation was continued at 37°C for 3 h. Nra was detected in whole‐cell extracts using immunoblot analysis with anti‐Flag mAb and HRP‐conjugated goat anti‐mouse IgG. Molecular mass standard sizes are indicated on the left. B. The delNra‐Mock and delNra‐p5′Flag‐Nra strains and an *nra* deletion mutant strain transformed with pMSP3545‐*nra* (delNra‐pNra) were cultured to the exponential phase at 37°C, then further cultured in the presence of nisin at the indicated concentrations at 37°C for 3 h. Cell wall fractions were immunoblotted with anti‐FctA and HRP‐conjugated goat anti‐mouse IgG.

To determine whether the intracellular Nra level has effects on T49 pilus expression, a nisin‐controlled expression (NICE) system was used to control transcriptional *nra* levels (Fig. 4). In the background of the *nra* deletion mutant, either *nra* or *flag*‐tagged *nra* was ectopically expressed at 37°C from a shuttle vector under control of the *nisA* promoter and NisKR two‐component system, which shows a response to extracellular nisin. Along with an increase in the nisin concentration and *nisA* promoter activity, transcription of the downstream gene, that is, *nra*, was upregulated. In the cytoplasmic fraction of a strain expressing 5′‐*flag*‐tagged *nra* (delNra‐p5′FlagNra), the level of Nra was increased in a nisin concentration‐dependent manner, whereas Nra was not detected in a control mock strain (Mock, Fig. 4A). Since the FctA49 production was not detected in the cell wall fraction of the mock strain, NisKR had no effect on pilus production in the absence of *nra*. In contrast, with an increased concentration of nisin, pilus expression was more readily detected in cell wall fractions of strains expressing *nra* (delNra‐pNra) or 5'‐*flag*‐tagged *nra* (delNra‐p5′FlagNra) (Fig. 4B). Therefore, a suprathreshold Nra protein level is essential for inducing the production of T49 pili.

### Heterologous expression of *nra* and *cpa* operon in *Lactococcus lactis* induces thermosensitive pilus production

**Figure 5:**
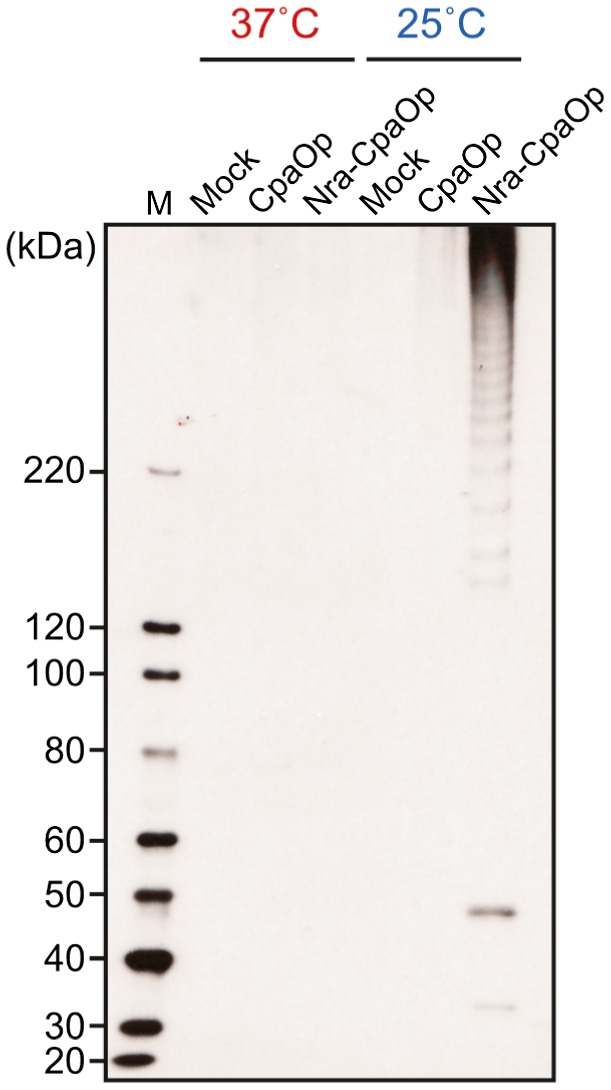
Heterologous expression of *nra* and *cpa* operon in *L. lactis* exhibits culture temperature‐dependent pilus production. Cell wall fractions of *L. lactis* strain NZ9000 transformed with an empty shuttle vector (Mock), or a vector harboring either the *cpa* operon (CpaOp) or *cpa* operon and *nra* (Nra‐CpaOp) were prepared from cells grown to the exponential phase at 37°C or 25°C (OD_600_ = 0.5). Cells were then immunoblotted with anti‐FctA antiserum. Molecular mass standard sizes are shown on the left. M, protein size marker.

To further examine the involvement of Nra in the thermosensitive process of pilus production, a series of *L. lactis* strains transformed with a mock vector (Mock), or a vector harboring either the *cpa* operon (CpaOp) or *nra*‐*cpa* operon (Nra‐CpaOp) were constructed, then FctA49 detection was performed in cell wall fractions of those strains cultured at 37 and 25°C (Fig. 5). When the *cpa* operon was expressed in the absence of *nra*, ladder bands were not detected in cell wall fractions at either temperatures. In contrast, when *nra* was cointroduced, FctA49 ladder bands were more readily detected at the lower culture temperature. Together with the findings noted above, these results suggest that Nra itself is a prerequisite for the thermosensitive pilus production in Nra‐positive FCT type 3 strains.

### Temperature‐dependent translational efficiency of *nra*

**Figure 6:**
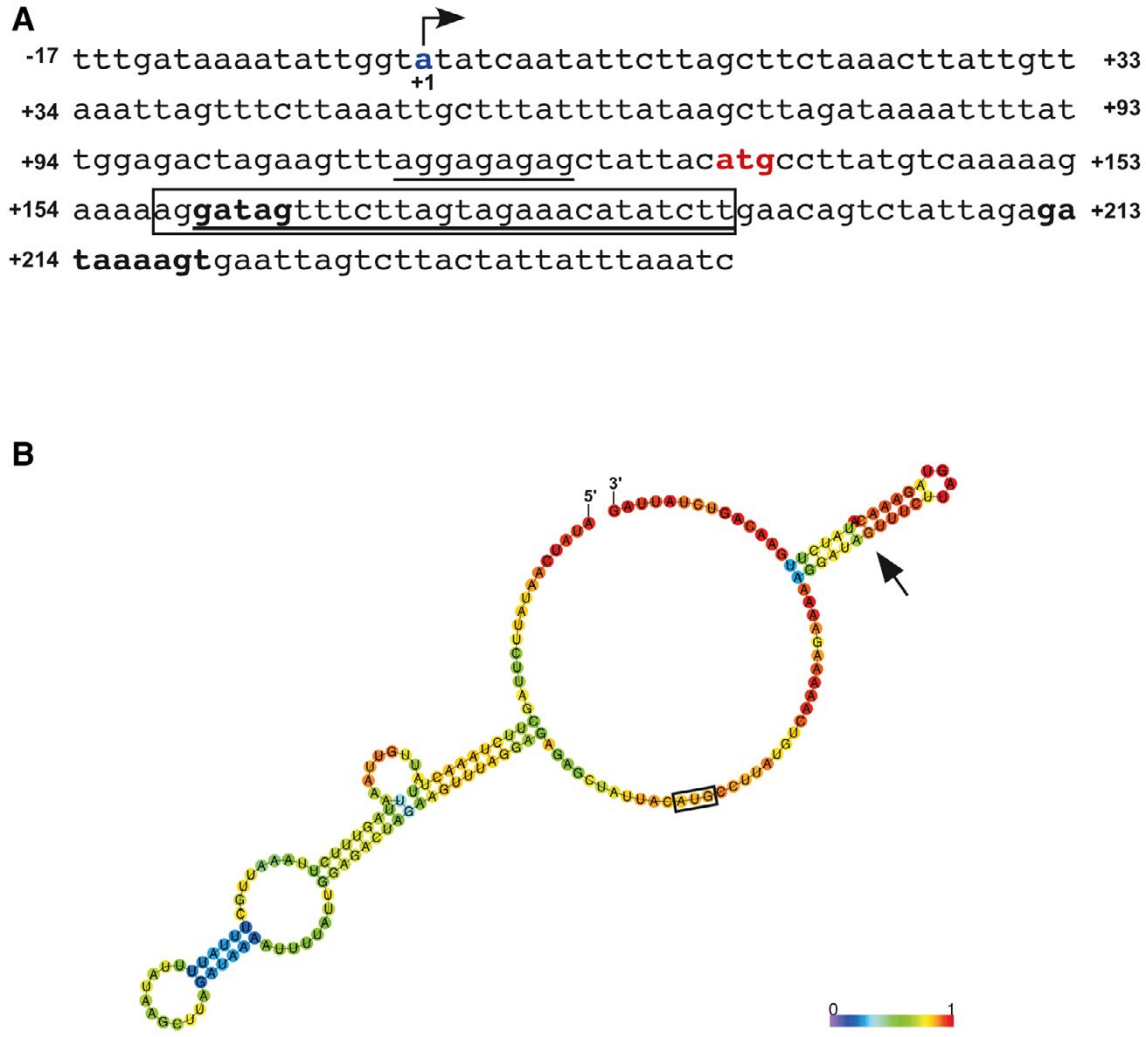
Putative stem‐loop structures in 5′ region of *nra* mRNA. A. Nucleotide sequence of the intergenic region of *nra* and *cpa* and 5′‐coding sequence of *nra*. The *nra* start codon is shown in red, with the ribosome binding sequence underlined. The transcriptional start site of *nra*, identified by 5′‐RACE analysis, is indicated in blue and defined as the +1 position for the strand encoding Nra. The boxed region corresponds to a putative stem‐loop structure within the coding region of *nra* mRNA. The four bases shown in bold are silent‐mutated bases in the mutant strain ‘4mut’, used as shown in Fig. 7. In addition, the 27 underlined bases and first 12 bases within the boxed region are deleted regions in the mutant strains ‘∆27’ and ‘∆12’ respectively. B. The predicted optimum structure of the 5' region of *nra* mRNA (+1‐+180) for generating minimum levels of free energy is shown. The translational start codon is indicated by a box and the stem‐loop structure within the protein‐encoding region by an arrow. The scale indicates base‐pairing probability, ranging from 0 (violet) to 1 (red).

To determine the transcriptional start site of *nra*, rapid amplification of cDNA ends (5'‐RACE) analysis was performed using total RNA isolated from strain 591 cultured at either 37°C or 25°C. Sequence analysis indicated that the transcriptional start site was present 115 bases upstream of the *nra* start codon (Fig. 6A) and not affected by the culture temperature. Utilizing this information, full‐length 5′‐*flag*‐tagged *nra* mRNA was synthesized *in vitro* and preincubated at 37°C or 25°C, then used for an *in vitro* translation reaction in an S30 *Escherichia coli* extraction system at 37°C or 25°C. The immunoblot analysis revealed that regardless of the preincubation temperature, Nra was efficiently synthesized at 25°C in these reactions (Fig. S2). Interestingly, slightly greater amounts of Nra were detected with *nra* mRNA preincubated at 37°C as compared to 25°C. These results indicated that the translational efficiency of *nra* mRNA is regulated by the temperature.

### Putative stem‐loop structure within *nra* mRNA promotes *nra* mRNA translation

Since the 5′ region of the mRNA structure was shown to most frequently be responsible for achieving temperature‐dependent post‐transcriptional control, the 5′‐region secondary structure of *nra* mRNA was computationally predicted using nucleotide sequences from the transcriptional start site (+1) to +180 (Fig. 6B). The results showed characteristic regions exhibiting extensive base‐pairing from position +6 to +113, while a stem‐loop structure was observed from position + 138 (+23 from start codon) to +166. The former base‐pairing region contains the SD sequence, which is puzzling in the view of thermosensitivity, because a decline in temperature strengthens base‐pairing in many cases and likely inhibits binding of mRNA to 30S ribosomes, thereby decreasing translation levels. The location of the latter stem‐loop structure has been shown to be 23 bases downstream of the AUG start codon, at a position outside of the region covered by the 30S subunits, in the presence of tRNA (Steitz, 1969; Dreyfus, 1988; Hüttenhofer and Noller, 1994; Yusupova *et al.*, 2001).

**Figure 7:**
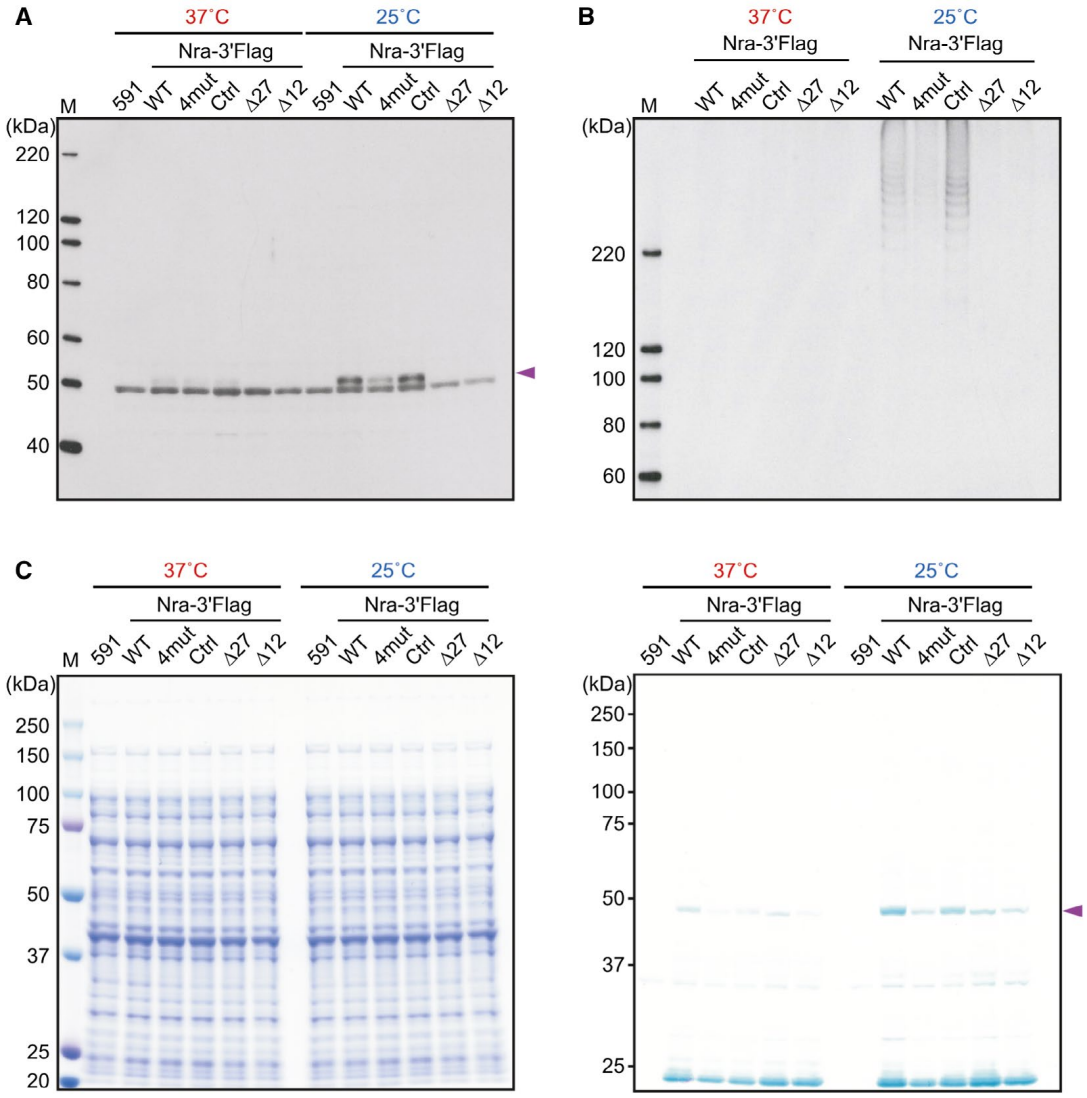
Stem‐loop structure within the coding region of *nra* mRNA necessary for efficient *nra* translation and pilus production. A. Whole‐cell extracts were prepared from the strain expressing 3′‐*flag* tagged *nra* (Nra‐3′Flag) and its derivative strains grown to the exponential phase at 37°C or 25°C (OD_600_ = 0.55), then immunoblotted using anti‐Flag mAb. Purple arrowhead indicates the band corresponding to Nra. B. FctA in cell wall fractions prepared from the indicated strains was detected by immunoblot analysis. C. *In vitro* transcription/translation of *nra* from the indicated strains was performed using a DNA fragment encompassing a region from the transcriptional start site to stop codon. Synthesized protein was separated by SDS‐PAGE, followed by either staining with Coomassie brilliant blue (left panel) or immunoblot analysis with anti‐Flag mAb (right panel). Purple arrowhead indicates the band corresponding to Nra. Molecular mass standard sizes are indicated on the left. M, protein size marker.

To examine the possible involvement of this stem‐loop structure in the thermosensitive translation of *nra* mRNA, four different mutant strains were constructed in the background of a strain expressing 3'‐Flag‐tagged Nra (WTflag). The loop structure was artificially melted by the introduction of chromosomal silent mutations into four bases of two codons encoding D9 and S10 (gat agt → gac tca) to generate strain ‘4mut’. As a control, we used a strain with silent mutations in codons encoding D23 and S25 to exclude the possibility of codon bias (4mut ctrl). Moreover, in‐frame deletion mutant strains lacking either the complete loop (∆27) or 5′ end of the loop (∆12) were also constructed. The predicted RNA structure indicated a small possibility that the ∆12 mutation would generate small stem‐loops at different positions, whereas no stem‐loop was predicted when the 4mut silent mutations and ∆27 mutation were introduced (Fig. S3). When bacteria were incubated at 37°C, no remarkable changes in levels of detected Nra were observed (Fig. 7A). In contrast, at 25°C, the Nra level in the 4mut and deletion mutant strains (∆27, ∆12) was decreased, as compared to that in the WTflag and 4mut ctrl strains (Fig. 7A). Concomitantly, the relative level of pili produced by each strain was correlated with the Nra level in a temperature‐dependent manner (Fig. 7B). However, since it is possible that the decreased level of pilus production by the deletion mutant strains ∆27 and ∆12 was caused by the loss of Nra regulatory function, we examined mRNA and protein levels of Nra and Cpa in *nra* deletion mutant strains overexpressing 3′‐*flag*‐tagged WT *nra* or a series of mutant *nra* under the *gyrA* promoter (Fig. S4). With the *gyrA* promoter, *nra* levels were increased by ~ 300‐fold as compared to that in Nra‐3′Flag strains cultured at 37°C or 25°C (Fig. S4A). Furthermore, the protein levels of Nra with the 4mut or ∆12 mutation were decreased as compared with those of WT Nra and Nra with the 4mut ctrl mutation, while the level of Nra with the ∆27 mutation decreased drastically (Fig. S4B), whereas *nra* expression levels were comparable to those of the other mutants (Fig. S4A). These results indicate that even with the 5′‐UTR sequence from the shuttle vector, the stem‐loop structure has an influence on the translation efficiency when *nra* is overexpressed by culturing at 37˚C. Comparative analyses of both protein and mRNA levels indicated that the ∆12 mutation led to a slightly decreased ability of Nra to promote the pilus production, while the ∆27 mutation caused complete loss of Nra regulatory function. Therefore, no detection of pili in the deletion mutant strains ∆27 and ∆12 (Fig. 7B) was likely due, at least in part in the case of ∆12, to the loss of Nra regulatory function and indicates that the Nra protein region encoded by the stem‐loop is important for the regulatory function.

As a reflection of those *in vivo* results, findings obtained with *in vitro* transcription/translation of DNA fragments encoding WT 3′‐Flag‐tagged Nra and mutant Nra confirmed that the stem‐loop structure within the coding region of *nra* mRNA is responsible for the temperature‐dependent translational efficiency of Nra (Fig. 7C). When the reaction was performed in the presence of protease inhibitors, no obvious change was observed (data not shown). Moreover, when WTflag was grown to the exponential phase at 25°C and divided into two cultures, and then further incubated at either 37°C or 25°C in the presence of rifampicin (1 mg/ml) and tetracycline (20 µg/ml) to halt both transcription and translation, there was no difference in Nra levels detected between 37 and 25°C (Fig. S5), excluding the possibility that Nra proteolysis is mainly attributable to the differential Nra level between 37 and 25°C.

To validate our data obtained with the serotype M49 strain and test the generality among FCT type 3 strains, in the background of a serotype M3 strain we examined Nra3 to determine if it also possesses a positive regulatory function with regard to the pilus production and whether the stem‐loop structure conserved as in *nra*3 is also involved in the translational efficiency of *nra* (Fig. S6). Utilizing an in‐frame *nra* deletion mutant strain and a strain expressing 3′‐*flag* tagged *nra* with the same chromosomal mutations described above for the 4mut and 4mut ctrl strains, positive effects of *nra* on thermosensitive pilus production and involvement of the stem‐loop structure in Nra level were also confirmed in the serotype M3 strain. These results indicated that Nra3 and its stem‐loop structure are also required for the thermosensitive T3 pilus production, while the stem‐loop structure of *nra* is fundamental for Nra thermosensitive translation and pilus production in FCT type 3 strains.

## Discussion

Adaptation by *S. pyogenes* strains to survive in specific human niches relies on their ability to fine‐tune the gene expression and translate a distinct subset of mRNA in response to diverse environmental cues, including temperature fluctuations. During the initial stage of *S. pyogenes* infection, strict regulation of transcription and translation of adhesin genes provides advantages for successful colonization. Among the numerous adhesins of *S. pyogenes*, pili are considered to mediate tropism in human tissues (Bessen, 2016). The remarkable genetic and antigenic variability of pili is demonstrated by the fact that the main subunit is responsible for the antigenicity of T serotyping. To fully understand the role of pili in the pathogenesis, their functions and expression modes need to be clearly understood. The present study revealed that in FCT type 3 strains, Nra positively affects the transcription of pilus‐related genes and that post‐transcriptional regulation of *nra* mRNA in a temperature‐dependent manner is required for generating the observed expression pattern of pilus‐related genes.

Thermoregulation of pilus expression in a serotype M49 strain was initially observed at the transcriptional level of *cpa* operon genes (Nakata *et al.*, 2009). In an earlier study, the response of *S. pyogenes* to temperature shifts was investigated at both transcriptional and translational levels, and results of microarray analysis with a serotype M1 strain demonstrated that approximately 9% of the genes were differentially expressed by at least 1.5‐fold at 29°C, relative to that observed at 37°C (Smoot *et al.*, 2001). As for virulence factors, temperature‐dependent changes in the expression levels of genes encoding the superantigen SpeA, EndoS glycosidase, α‐amylase and capsule amounts have also been reported (Xu and Collins, 1996; Nakamura *et al.*, 2004; Kang *et al.*, 2012). Furthermore, as a means of post‐translational thermoregulation of protein functions, temperature shift modulates the conformation of the M protein family and has effects on protein functions (Akerström *et al.*, 1992; Qiu *et al.*, 2019). Thus, it is likely that gene expressions, protein levels and protein functions in *S. pyogenes* are profoundly altered in response to changes in temperature. However, more integrated and detailed analysis is needed to better reveal the precise mechanism underlying the temperature perception and response system of *S. pyogenes*, as well as its association with pilus thermoregulation and virulence.

The biological significance of thermosensitive pilus production in FCT type 3 strains remains unclear and an important question is why other FCT type strains have not adopted this mode of pilus production. Blood survival test findings in the present study indicate that pilus production confers serotype M49 strains an ability to survive and proliferate in human blood. It has also been reported that pili produced by M2 (FCT type 6) and M53 (FCT type 3) strains promote bacterial survival in human blood (Rouchon *et al.*, 2017; Tsai *et al.*, 2017). In contrast, constitutive pilus production in an M1 strain (FCT type 2) was found to promote bacterial adherence to epithelial cells, but reduced survival rates in human blood and attenuated virulence in both *Galleria mellonella* larvae and mouse infection models (Crotty Alexander *et al.*, 2010; Loh *et al.*, 2013). Although these findings highlight remarkable differences in terms of either pilus function or sensitivity toward the human immune system, implications related to the differential pilus production mode cannot be simply explained. One possible reason for the thermosensitive pilus production is that the evolution of FCT type 3 *S. pyogenes* has provided for tight control of the expression of pilus‐related genes to prevent futile energy consumption, as inferred from previous studies stating that the production and secretion of virulence factors is an energy‐consuming process (Cornelis *et al.*, 1998; Shao, 2008). Another possibility is that the production of immunogenic pilus proteins results in a fitness cost, due to the selective pressure of human immune response. The difference in the pilus production mode between FCT type 3 and other types may be attributable to functional and antigenic differences of each type of pilus and the resultant degree of relative contribution to virulence and fitness. Since the pilus gene expression by FCT type 3 and other types is regulated by distinct regulators, the involvement of other factors whose expression is also regulated by the respective regulators might contribute to variabilities in the pilus production mode.

The antigenicity of major pilus subunits is a determinant for T serotyping (Falugi *et al.*, 2008). The present findings revealed that FCT type 3 strains are unable to produce detectable pili when cultured at the conventional temperature of 37°C. However, a significant level of pilus expression was induced at a lower temperature. Interestingly, the pilus expression pattern observed in FCT type 3 strains is reminiscent of culture conditions commonly used for T‐typing. According to the general protocol for T serotyping, it is recommended to perform the culturing process at 30°C. Thus, a portion of the molecular mechanism associated with this temperature constraint was elucidated by the present findings.

Presently, there is no vaccine against *S. pyogenes* commercially available. Protective immune responses were found to be evoked by the immunization of pilus proteins in a mouse infection model and pili can be displayed outside the peptidoglycan and capsule layers, thus pilus proteins have gained attention as vaccine antigens (Mora *et al.*, 2005). However, as observed with the development of multivalent vaccines for M protein hypervariable regions, the antigenic variability of pilus proteins poses a challenge for the development of either a universal or broad‐spectrum vaccine. As indicated in the present results, pilus production by FCT type 3 *S.pyogenes* is decreased during the dissemination period, when the surrounding temperature around bacterial cells increases. Although this is not applicable to other FCT types, including at least FCT types 1, 2, 4, 5, 6 and 7, as shown in this study, it might be necessary to consider this aspect when attempting to develop effective pilus‐based vaccines.

Temperature fluctuations in the environment can change the properties of bacterial surface proteins, thereby affecting molecular interactions between bacteria and their host. In the present study, an M49 strain was able to adhere to human keratinocytes in a pilus‐dependent manner only when the initial bacterial culture was at a lower temperature. Results of thermal mapping of the human airway and skin surface have shown that the temperature at these sites fluctuates in a range between the high 20s and lower 30s and is considerably affected by various factors, including ambient air temperature (Abe *et al.*, 1980; McFadden *et al.*, 1985; Rouadi *et al.*, 1999; Keck *et al.*, 2000; Smith *et al.*, 2010). Interestingly, epidemiological studies have shown that episodes of *S. pyogenes* infections are predominant from the early winter to early spring seasons, even though the seasonality pattern in different geographic areas is varied (Kaplan *et al.*, 2001; Muller *et al.*, 2003; Rubinstein *et al.*, 2005; Danchin *et al.*, 2007; Lamagni *et al.*, 2008; Olafsdottir *et al.*, 2014; Smit *et al.*, 2015; Hervás *et al.*, 2016; Nelson *et al.*, 2016). Findings obtained in the present study indicate that thermoregulation of pili in FCT type 3 strains is a factor contributing to *S. pyogenes* colonization in initial infection sites, that is, the throat and skin, and that the temperature should now be considered as an important parameter in studies of the interactions between *S. pyogenes* and host cells.

Although the molecular basis for the tissue tropism of *S. pyogenes* strains is not completely understood, three major *emm* pattern groups, known as A‐C, D and E, based on sequence differences in the conserved 3′ end of *emm* and flanking *emm*‐like genes, such as *mrp* and *enn*, have been reported to have significant associations with pharyngitis and impetigo (Bessen, 2016). *emm* pattern A–C and D strains have been designated as specialists specifically targeting the throat and skin, respectively, whereas *emm* pattern E strains have been isolated nearly equally from the throat and skin samples, thus are designated as generalists. Kratovac *et al*. reported that among 113 strains of 111 distinct *emm* types, pattern A–C strains represented approximately 47% of pharyngitis isolates and 8% of impetigo isolates, whereas pattern D strains represented approximately 50% of impetigo isolates and less than 2% of pharyngitis isolates (Kratovac *et al.*, 2007). That report also noted that among 39 *emm* types of *nra*‐positive FCT types 3 and 8, 32 of 39 types (88.8%) represent pattern D. The corresponding *emm* patterns of the other seven *emm* types are *emm*3 (A–C), *emm*5 (A–C), *emm*18 (A–C), *emm*49 (E), *emm*73 (E), *emm*94 (E) and *emm*117 (E). Therefore, the majority of FCT types 3 and 8 strains can be classified as skin specialists, raising the possibility that thermosensitive pilus production by these FCT type strains is required for skin infection to occur. Elucidation of this association awaits further research regarding the interactions of pilin molecules with human host factors.

Environmental temperature induces changes at both transcriptional and post‐transcriptional/translational levels during prokaryotic gene transcription and translation. Thermosensing arises from the structural lability of a wide variety of molecules, ranging from nucleic acids and lipids to proteins. In particular, the diversity of the underlying mechanisms of post‐transcriptional regulation via mRNA can be attributed to the remarkable plasticity of RNA due to the presence of thermally labile base pairs. A wide variety of pathogenic bacteria exploit RNA thermosensors to facilitate adaptation to the surrounding environment of host niches and establish infection (Konkel and Tilly, 2000). The present results revealed that the thermosensitive regulation of *nra* mRNA translation is responsible for the thermoregulation of T49 pilus production. It is thus considered that *nra* thermosensitivity has great potential for shaping the expression level of pilus‐related genes, leading to enhanced colonization in the host.

Translational efficiency is dependent on the levels of initiation and elongation that occur during translation (Kudla *et al.*, 2009; Cannarozzi *et al.*, 2010; Tuller *et al.*, 2010; Osterman *et al.*, 2013). The latter is determined by codon usage and tRNA abundance (Fredrick and Ibba, 2010), while the former is primarily dependent on both mRNA secondary structures and *cis*‐elements, including the SD sequence and enhancers that promote binding of mRNA to the 30S ribosomal subunit (de Smit and van Duin, 1990; Brock *et al.*, 2007; Sharma *et al.*, 2007; Yang *et al.*, 2014). Recently, Jagodnik *et al.* reported that a stem‐loop structure located around the position +20 from the start codon of *E. coli fepA* mRNA caused initiation of the translation process at the ternary complex, which was independent of the nucleotide sequence, though dependent on the distance between the start codon and stem‐loop (Jagodnik *et al.*, 2017). As a result, they proposed a ‘starting block’ mechanism, through which the stem‐loop blocks the 30S ribosomal subunit from sliding onto mRNA without establishing a translation initiation complex, leading to optimized positioning of the 30S subunit and an increased rate of translation initiation. Additionally, Paulus *et al*. reported that the introduction of a 7‐bp stem‐loop at position +19 improved heterologous gene translation (Paulus *et al.*, 2004). In line with those reports and proposed models, the predicted stem‐loop structure of *nra* might be responsible for the thermosensitive translational control of *nra* mRNA, though it is likely that other *cis*‐elements of *nra* mRNA, such as 5'‐UTR, function cooperatively for thermoregulatory translation. A feasible scenario is that the stem‐loop structure is prone to undergo melting at the core body temperature, while a relatively lower temperature reflecting the initial infection site provokes stable base‐pairing, allowing *nra* mRNA to set the 30S subunit in an appropriate position and augment the subsequent translational process.

The thermosensing property of *nra* mRNA is distinct from that of previously reported common thermoresponsive RNA structures, in which alternative base‐pairing structures are formed in response to changes in temperature, to either enhance the accessibility of RBS or preclude ribosome binding (Kortmann and Narberhaus, 2012). In this study, we were unable to exclude the possibility of post‐translational control of Nra, such as temperature‐dependent alteration in protein conformation and multimeric configurations, or post‐transcriptional dual regulation with noncoding RNAs, which reportedly interact with bacterial factors, including transcriptional regulators (Hurme *et al.*, 1997; Grimshaw *et al.*, 2003; Loh *et al.*, 2009; Saita *et al.*, 2015). Results revealing the details related to the molecular mechanisms underlying the temperature perception system of *nra* mRNA, that is, the temperature‐relevant regulatory element of *nra* mRNA, will help to identify potential interactions with unknown factors and provide a rationale for defining the *nra* thermometer.

In summary, the thermosensitive pilus production was observed in FCT type 3 strains of *S. pyogenes* and shown to be due to temperature‐dependent translation of the Nra transcriptional regulator. Thermoregulated pilus production provided a serotype M49 strain with abilities to adhere to human keratinocytes and survive in human blood. Additionally, the stem‐loop structure within the coding region of *nra* mRNA was shown to be required for the temperature‐dependent post‐transcriptional control of the thermosensitive translation of *nra* mRNA. This is the first report providing evidence of an mRNA‐based thermometer in *S. pyogenes* that, interestingly, does not resemble classical 5′‐UTR thermosensors, but rather exists deep inside mRNA. These findings add a completely new and previously unrecognized level of regulation into the network of existing concepts of virulence factor gene regulation in *S. pyogenes*. Additional elucidation of the entire temperature‐dependent *nra* regulon as well as *in vivo* infection experimental results will enable precise determination of the role of the *nra* thermometer in the ecology and pathogenesis of FCT type 3 *S. pyogenes*. It is tempting to speculate that translational activation through this type of stem‐loop structure is restricted to a distinct subset of mRNAs, whose translational products are required for the adaptation to the host environment and colonization at the initial stage of infection. Future analyses focusing on the effective elucidation of the complex environment‐perception system of this important human pathogen are necessary.

## Experimental procedures

### Bacterial strains and culture conditions

*S. pyogenes* strains were selected from the stock culture collection of the Department of Oral and Molecular Microbiology, Osaka University Graduate School of Dentistry (Osaka, Japan) and Institute of Medical Microbiology, Virology and Hygiene, University of Rostock (Rostock, Germany) (Köller *et al.*, 2010; Murakami *et al.*, 2002). A serotype M49 strain 591 was isolated from a subject with skin infection. *S. pyogenes* clinical isolates and mutant strains were cultured in Todd‐Hewitt broth (Becton Dickinson) supplemented with 0.2% yeast extract (Becton Dickinson) (THY medium) at ≤37°C in an ambient atmosphere. The *E. coli* strains XL10‐gold (Stratagene) and TOP10 (Life Technologies) served as hosts for derivatives of plasmids pSET4s, pAT18 and pMSP3545 (Trieu‐Cuot *et al.*, 1991; Bryan *et al.*, 2000; Takamatsu *et al.*, 2001). *E. coli* strains were cultured in Luria‐Bertani (LB) medium at 37°C. *L. lactis* strain NZ9000 was cultured in M17 medium (Gibco) supplemented with 0.5% glucose (WAKO) (MG medium). For the selection and maintenance of mutant strains, antibiotics were used to supplement bacterial cultures at the following concentrations: ampicillin (Sigma‐Aldrich), 100 µg/ml for *E. coli*; spectinomycin (Wako), 100 µg/ml for both *E. coli* and *S. pyogenes*; and erythromycin (Sigma‐Aldrich), 300 µg/ml for *E. coli* and 1 µg/ml for *L. lactis* and *S. pyogenes*.

### DNA cloning

Chromosomal DNA from *S. pyogenes* was purified using a DNA extraction kit (Takara). Amplification of DNA fragments was performed with Phusion Hot Start DNA polymerase (Finnzymes), Tks Gflex and *ExTaq* DNA polymerases (Takara). Plasmid DNA was purified using a plasmid purification kit (Macherey‐Nagel). T4 DNA ligase (Takara) was used for ligating DNA fragments. Transformation of *E. coli*, *L. lactis* and *S. pyogenes* was performed as previously described (Nakata *et al.*, 2011; Morita *et al.*, 2014). Restriction enzymes were purchased from New England Biolabs (NEB).

### Preparation of mouse antisera against FctA proteins

DNA fragments encoding mature FctA without putative signal sequences and C‐terminal sorting signals from M1, M3 and M49 strains were PCR‐amplified and cloned into pQE30 (Qiagen). These plasmids were transformed into *E. coli* strain XL10‐Gold and N‐terminally His‐tagged FctA proteins were expressed in the presence of 1 mM isopropyl β‐*D*‐1‐thiogalactopyranoside. Recombinant FctA proteins were affinity purified from soluble fractions of whole‐cell extracts using Ni‐NTA resin, according to the manufacturer’s instructions. Eluates were dialyzed against phosphate‐buffered saline (PBS, 137 mM NaCl, 10 mM Na_2_HPO_4_, 2.7 mM KCl, 1.76 mM KH_2_PO_4_, pH7.4) and concentrated using an Amicon Ultra centrifugation unit (Millipore). Protein concentrations were determined using a BCA Protein Assay Kit (Thermo Fisher Scientific).

Mouse antisera against FctA1, FctA3 and FctA49 were raised by immunizing 5‐week‐old female BALB/c mice with the purified recombinant proteins. Briefly, the mice were intradermally administered an emulsion of 100 µg of recombinant proteins and TiterMax Gold adjuvant (CytRx). After 2 weeks, five boosts with 100 µg of recombinant proteins were repeatedly administered with the same adjuvant at 1‐week intervals. Whole blood was collected from the orbital sinus and antiserum was purified for immunoblot analysis, as previously described (Nakata *et al.*, 2009).

### Immunoblot analysis

To detect the pilus major subunit FctA, *S. pyogenes* and *L. lacti*s cells were grown at 37°C or 25°C until the late exponential phase (OD_600_ = 1.0 for *S. pyogenes*; 0.5 for *Lactococcus*), then washed with PBS and suspended in protoplast buffer containing 0.1 M KPO_4_ (pH 6.2), 40% sucrose, 10 mM MgCl_2_, complete EDTA‐free protease inhibitors (Roche) and 250 µg/ml *N*‐acetylmuramidase SG (Seikagaku Biobusiness). The suspension was incubated at 37°C for 3 h and protoplasts were sedimented by centrifugation at 20,000 × *g* for 20 min. Proteins in the supernatant were separated in a 5–12% acrylamide gel by sodium dodecyl sulfate‐poly‐acrylamide gel electrophoresis (SDS‐PAGE) and blotted onto polyvinylidene difluoride (PVDF) membranes. The membranes were blocked overnight using a casein‐based blocking reagent (Megmilk Snow Brand) at 4°C and incubated at room temperature (RT) for 1 h with mouse antisera diluted 1:2,000 in Tris‐buffered saline (TBS) containing 0.2% Tween 20 (TBST). After washing three times with TBST, each membrane was incubated at RT with horseradish peroxidase (HRP)‐conjugated anti‐mouse IgG antibody (Cell signaling), diluted at 1:2,000 in TBST for 1 h. The membranes were then washed three times and developed using the Pierce western blotting substrate (Thermo Scientific) or 3,3′,5,5′‐tetramethyl‐benzidene colorimetric substrate (MOSS).

### Fluorescence‐activated cell sorter analysis of pilus expression

*S. pyogenes* strains were grown to the exponential phase (OD_600_ = 0.6) at either 37˚C or 25˚C, then washed twice in PBS and blocked with a PBS‐based blocking buffer containing 2% bovine serum albumin and 5% casein‐based solution (Megmilk Snow Brand) for 30 min. Next, bacterial cells were incubated with anti‐FctA mouse antiserum (1:500 diluted). Nonimmunized mouse serum was used as a negative control. Cells were washed three times with PBS and incubated with fluorescein isothiocyanate (FITC)‐conjugated goat anti‐mouse IgG (1:500 dilution) for 1 h at RT, followed by three washes with PBS. The cells were then resuspended in PBS and pilus expression was examined using a flow cytometer, at a collection rate of 100,000 events. Data obtained were analyzed with FlowJo software (Tree Star, Ashland, OR).

### Eukaryotic cell adherence assay

Human keratinocyte HaCaT cells were maintained in Dulbecco's modified Eagle's medium (DMEM, WAKO) supplemented with 10% fetal bovine serum (FCS, SAFC Biosciences) and 20 µg/ml of gentamicin, as previously reported (Sumitomo *et al.*, 2011). Cells were seeded at 1 × 10^5^ cells per well in a 24‐well culture plate (IWAKI) and cultured for 1–2 days at 37°C, in the presence of 5% CO_2_. One day before infection, cells were washed with DMEM and the culture medium was replaced with DMEM/10% FCS without gentamicin. *S. pyogenes* strain 591 and an isogenic strain with the *cpa* operon deleted (∆Cpa) (Nakata *et al.*, 2009) were grown overnight at both 37°C or 28°C and used for the assays. Confluent HaCaT cell monolayers were infected with the *S. pyogenes* strains at a multiplicity of infection (MOI) of 10 for 2 h. Infected cells were washed three times with PBS and lysed with distilled water. Serial dilutions of the lysate were plated on THY agar plates. After incubating the agar plates at 37°C for 24 h, bacterial colonies were counted and colony‐forming units (CFUs) were determined. The bacterial adherence rate was calculated as a percentage of inoculated CFUs.

### Blood survival assay

Strains 591 and ∆Cpa were cultured at 25°C until the late‐exponential phase (OD_600_ = 1.0). Bacterial cells were washed with PBS and resuspended in it, then their density was adjusted to 4,000 CFU/ml. Next, 480 µl of heparinized human blood collected from a healthy donor was mixed with 20 µl of bacterial suspension containing 200 CFUs. Following 1 or 2 h of incubation at 37°C on a rotary mixer, the mixture was plated onto THY agar plates. After incubating cells at 24 h, grown colonies were counted and the multiplication factor was calculated as the percentage of inoculated CFUs.

### Construction of mutant strains

Strain delNra, an in‐frame deletion mutant strain of the *nra* gene, was constructed in the background of the WT strain using the temperature‐sensitive shuttle vector pSET4s (Nakata *et al.*, 2011). A pSET4‐NraKO plasmid harboring the DNA fragment, in which upstream and downstream regions of *nra* were linked by overlapping PCR, was electroporated into strain 591 and grown in the presence of spectinomycin. The plasmid was then integrated into the chromosome via first allelic replacement at 37˚C, after which it was cultured at 28°C without antibiotics to induce the second allelic replacement. The deletion of *nra* was confirmed by site‐specific PCR using purified genomic DNA.

A deletion mutant of *nra* was chromosomally complemented with a DNA sequence encoding 5′‐flag‐tagged Nra. First, the *nra* fragment was amplified using the primers NraF/Not and NraR/Kpn and inserted into p3XFLAG‐Myc‐CMV‐26 (Sigma‐Aldrich) via *Not* I and *Kpn* I sites to generate pFlag‐Nra. Utilizing pFlag‐Nra as a template, the DNA fragment encoding Flag‐tagged Nra was amplified using the primers nra‐flag‐F2 and nra‐flag‐R2. The fragment was PCR‐linked with two other DNA fragments containing the regions upstream and downstream of *nra*. The generated fragment was cloned into pSET4s and the resultant plasmid, pSET4‐5′Flag‐Nra, was transformed into the *nra* deletion mutant strain. This enabled chromosomal complementation of a fragment encoding 5′‐Flag‐tagged Nra to generate a 5′Flag‐Nra strain. To construct a deletion mutant chromosomally complemented with DNA encoding 3′‐Flag‐tagged Nra, two PCR‐amplified fragments containing the regions upstream and downstream of *nra* and a fragment of DNA encoding 3′‐Flag‐tagged Nra were linked via overlapping PCR, then cloned into pSET4 to generate pSET4‐Nra‐3′Flag. Finally, the plasmid was transformed to generate the Nra‐3′Flag strain, as described above.

To achieve the inducible expression of *nra* in the delNra strain, the NICE system was utilized. *nra* was expressed from the *PnisA* promoter, using pMSP3545, in which the NisRK two‐component system and *nisA* promoter regions were encoded (Bryan *et al.*, 2000). *PnisA* promoter DNA was amplified by PCR using the primers nrapmspF1 and nrapmspR1, and fused with a DNA fragment encompassing the region from the transcriptional start site to the putative terminator region of *nra*, which was amplified with the primers nrapmspF2 and nrapmspR2 using genomic DNA of strain 591 or strain 5'Flag‐Nra. The fused product was digested with *BamH* I and *Pst* I and cloned into pMSP3545 via the *Bgl* II and *Pst* I sites to generate the constructs pMSP‐Nra and pMSP‐5'Flag‐Nra. These plasmids were transformed into the delNra strain to generate the strains delNra‐pNra and delNra‐p5’FlagNra, which expressed *nra* and *flag*‐tagged *nra*, respectively, under the control of the extracellular nisin concentration. Empty pMSP3545 was also transformed to obtain a delNra‐mock strain, used as a negative control.

For heterologous expression of the *cpa* operon and *nra* in *L. lactis*, the DNA fragment harboring the entire *cpa* operon and *nra* was amplified using the primers OperonNraF and OperonR and chromosomal DNA from strain 591 as a template. The amplicon was inserted into pAT18 via the *BamH* I and *Pst* I sites to generate pAT‐Op‐Nra. To delete the *nra* gene from pAT‐Op‐Nra, inverse PCR was performed using pAT‐Op‐Nra as a template DNA. Following digestion with *Sal* I, the DNA fragment was self‐ligated to generate pAT‐Op. The empty pAT18 plasmid or constructed plasmids were transformed into *L. lactis* strain NZ9000 to generate Mock, CpaOp and Nra‐CpaOp strains.

For the chromosomal introduction of silent or deletion mutations into the *nra* region corresponding to the stem‐loop structure, pSET4s was also used to generate the mutant strains 4mut, Ctrlmut, del27 and del12.

All primers utilized are listed in Supplemental Table S2. All plasmid constructs, as well as deletion or introduction of genes, were confirmed by DNA sequencing and site‐specific PCR.

### 5′ Rapid amplification of cDNA ends

The 5′‐RACE analysis of *nra* was conducted using the 5′‐Full RACE Core Set (Takara), according to the manufacturer's instructions. Total RNA was isolated from strain 591 grown to the late exponential phase (OD_600_ = 0.9), as previously described (Nakata *et al.*, 2011). cDNA was synthesized from total RNA, using 5′‐phosphorylated primer RTnra5P and AMV reverse transcriptase. After eliminating RNA from the DNA‐RNA complex using RNase H, single‐stranded cDNA was concatenated using T4 RNA ligase. Then, the first PCR was performed with the primer set S1nra/A1nra using cDNA as a template. The amplified PCR product was then subjected to second PCR reactions as a template DNA along with the primer set S2nra/A2nra. PCR products were electrophoresed and gel‐purified using the NucleoSpin Extract II kit (Macherey‐Nagel). Purified fragments were TA‐cloned into the pGEM‐T vector (Promega). The resultant plasmid was sequenced with M13 primers and the BigDye Terminator v3.1 Cycle Sequencing Kit (Applied Biosystems), according to the manufacturer’s instructions, to confirm the transcriptional start site of *nra*.

### NICE of *nra*

The delNra‐Mock, delNra‐pNra and delNra‐p5'FlagNra strains were grown at 37˚C to the mid‐exponential phase (OD_600_ = 0.4) in 10 ml of THY, then nisin was added at indicated concentrations and incubation was further performed for 3 h. To examine Nra levels, delNra‐p5'FlagNra was washed two times with PBS and resuspended in 100 µl of lysis buffer containing 50 mM Tris (pH 8.0), 10 mM MgCl_2_, 10 units of phage lysin, 250 units/ml TurboNuclease (Accelagen) and an EDTA‐free complete protease inhibitor cocktail. The mixture was incubated at 37°C for 1 h and vortexed intermittently. After performing centrifugation at 20,000 × *g* for 10 min, the supernatant was used for immunoblot analysis. Flag‐tagged Nra was detected using the anti‐Flag tag monoclonal antibody M2 (Sigma‐Aldrich) and HRP‐conjugated anti‐mouse IgG antibody (Cell signaling). To detect FctA after incubation with nisin, cells were processed as described above.

To examine Nra levels in the 4mut, 4mut Ctrl, del27 and del12 strains, cells were grown to the mid‐exponential phase (OD_600_ = 0.55), then washed with PBS and resuspended in PBS supplemented with complete EDTA‐free protease inhibitor and 250 µg/ml *N*‐acetylmuramidase SG. Next, the cells were disrupted with glass beads (Sigma‐Aldrich), using a Mixer Mill MM300 (Qiagen) at a maximum speed for 10 min. After centrifugation at 20,000 × *g* for 10 min, the supernatant was used for the immunoblot analysis.

### Real‐time RT‐PCR

Utilizing a Transcripter high fidelity cDNA synthesis kit (Roche), cDNA was synthesized from three independent total RNA samples prepared from strain 591 and its isogenic mutant strains grown at 37°C or 25°C to the late exponential phase (OD_600_ = 1.0). Primers used were designed with Primer Express Software (version 3.0; Applied Biosystems) and are listed in Supplemental Table S2. RT‐PCR amplification was performed with SYBR Green and the ABI StepOne^TM^ System (Applied Biosystems). Relative expression levels were calculated using the ∆∆*C*_T_ method. *gyrA* expression data served as an internal control that was also used as a control to examine the gene expression levels of *S. pyogenes* cultured at 25–37°C (Smoot *et al.*, 2001; Kang *et al.*, 2012).

### *In vitro* transcription/translation

*In vitro* transcription/translation was conducted using a DNA fragment amplified with the primers nrainvitroR and nrainvitroFtac, which were prepared using genomic DNA from the 4mut, Ctrlmut, del27 and del12 strains. PCR products, including the *Ptac* sequence, were purified using a NucleoSpin Extract II kit (Macherey‐Nagel) and then subjected to an *E. coli* S30 Extract System for Linear Templates (Promega). The reactions were conducted at 37°C or 25°C for 2 h. Proteins were precipitated with acetone and suspended in SDS‐PAGE sample buffer. After boiling for 2 min, immunoblot analysis using anti‐Flag mAb was performed to detect Nra.

### Prediction of *nra* mRNA secondary structure

CentroidFold analysis was performed according to the instructions presented on the associated web server (http://rtools.cbrc.jp/centroidfold/) with the 180‐base long RNA sequence from the *nra* mRNA 5′ region (Sato *et al.*, 2009). CONTRAfold was used as the inference engine, with the weight of the base pairs was set to 2^2^.

### Statistical analysis

Statistical analysis was conducted using the GraphPad Prism software package (ver. 7.0). The significance of differences between the mean values of two groups was evaluated using a Mann‐Whitney *U* test. The comparison of mean values for multiple groups was performed using the Tukey's comparison test with ANOVA. A confidence interval with a *P *< 0.05 was considered to indicate a significance.

### Ethics statement

The human blood sampling protocol was approved by the institutional review board of Osaka University Graduate School of Dentistry (approval no. H26‐E43).

## Author contributions

MN designed the study. MN and TS performed experiments to acquire data. MN, TS, NP, BK and SK contributed to the interpretation of data and writing the manuscript.

## Conflicts of interest

The authors have no conflicts of interest to declare in regard to this study.
