# Supplementary material for: Thermosensitive pilus production by FCT type 3 Streptococcus pyogenes controlled by Nra regulator translational efficiency
Source: Mol Microbiol. 2019 Nov 11;113(1):173–89. doi: 10.1111/mmi.14408 (PMC7079067; doi:10.1111/mmi.14408)
Supplement: Supplementary file 1 [file MMI-113-173-s001.pdf]

Supplemental Information

Thermosensitive pilus production by FCT type 3 *Streptococcus pyogenes* controlled by Nra regulator translational efficiency

Masanobu Nakata, Tomoko Sumitomo, Nadja Patenge, Bernd Kreikemeyer, and Shigetada Kawabata

Supplemental Figure 1.

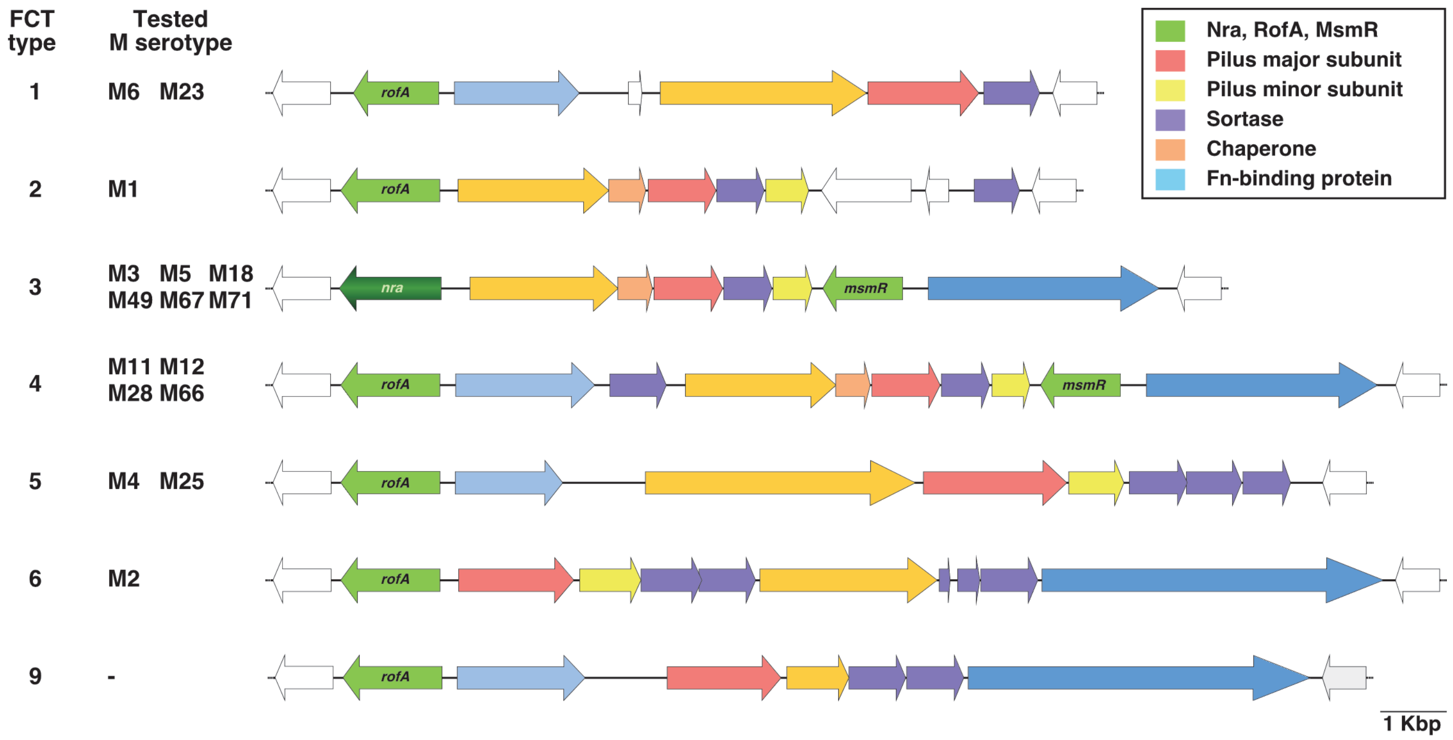

Supplemental Figure 1. Genetic map of FCT chromosomal region

Based on available genome sequences and previously reported data, the genetic content for a representative strain of each FCT type is shown (Kratovac et al., 2007; Falugi et al., 2008). Genome sequences for FCT type 7 and 8 strains are unavailable, thus genetic maps for those are not included. Tested M serotypes belonging to each FCT type are also shown. Major and minor subunit genes of pili are shown in red and yellow, respectively. Genes encoding the chaperon and transpeptidases required for pilus biogenesis are shown in orange and purple, respectively. Genes encoding fibronectin-binding proteins, including PrtF1, PrtF2, Pfbp, and FbaB, are shown in blue, and those encoding transcriptional regulators, including RofA, Nra, and MsmR, in green. Notably, *nra* was found present in FCT types 3 and 8. Sequence accession numbers and strains for each FCT and M types are as follows: type 1, M6, NC_006086, and MGAS10394; type 2, M1, NC_002737, and SF370; type 3, M3, NC_004606, and SSI-1; type 4, M12, AF447492, and A735; type 5, M4, NC_008024, and MGAS10750; type 6, M2, NC_008022, and MGAS10270; and type 9, M75, STAB14018, CCP014542.1.

Supplemental Figure 2.

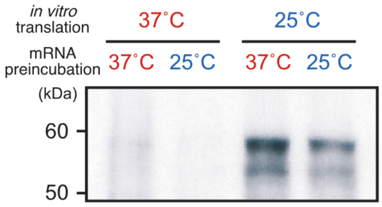

Supplemental Figure 2. Translation of *nra* mRNA promoted by decrease in temperature

5'-*flag*-tagged *nra* mRNA was synthesized by in vitro transcription, using an MAXIscript® Kit (Ambion), according to the manufacturer's instructions. The *nra* fragment was PCR-amplified using genomic DNA from the 5'Flag-Nra strain and primers, one of which was designed to include the minimal sequence recognized by T7 RNA polymerase. The amplicon was purified with a NucleoSpin Extract II kit (Macherey-Nagel) and used as template DNA for run-off transcription. The purity and size of the transcript were confirmed by electrophoresis in TBE buffer (100 mM Tris, 100 mM boric acid, 20 mM EDTA, pH 8.0), along with a denaturing 5% polyacrylamide gel containing 8 M urea and ethidium bromide staining. *In vitro* synthesized *nra* mRNA was pre-incubated at 37˚C or 25˚C for 30 min, then mRNA at 10 µg was used for each translation reaction with the *E. coli* S30 Extract System for Linear Templates (Promega), according to the manufacturer's instructions. Reactions were allowed to occur at 37˚C or 25˚C for 1 h. Proteins were precipitated with acetone and suspended in SDS-PAGE sample buffer. After boiling the samples for 2 min, immunoblot analysis was performed to examine the translational efficiency of *nra* mRNA at 37˚C and 25˚C. Synthesized Flag-tagged Nra was detected using anti-Flag tag mAb. Molecular mass standard sizes are shown on the left.

Supplemental Figure 3.

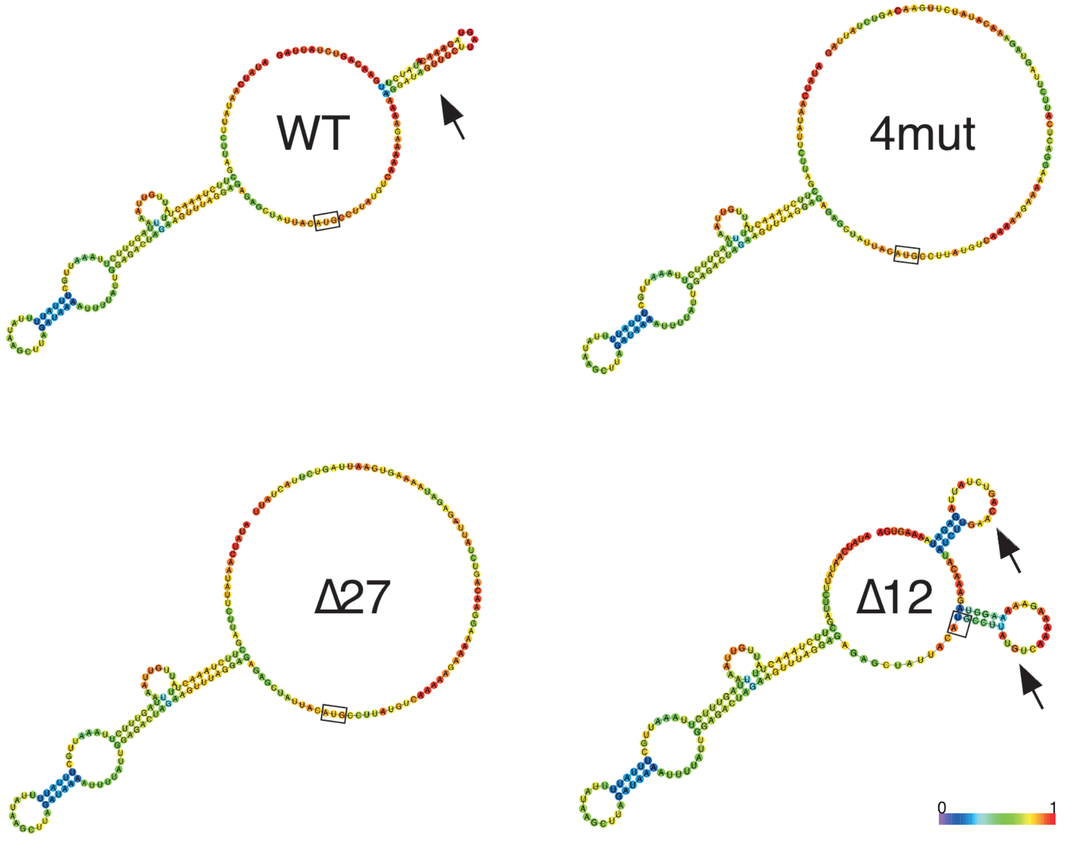

Supplemental Figure 3. Predicted structure of 5' region in wild-type and mutant *nra* mRNA

Predicted optimum structures of the 5' region of wild-type and mutant *nra* mRNA (+1-+180) are shown, in which the gene was either silent-mutated or deleted. The translational start codon is indicated by a box and the stem-loop structure within the protein-encoding region by arrows. Scale indicates base-pairing probability, ranging from 0 (violet) to 1 (red).

Supplemental Figure 4.

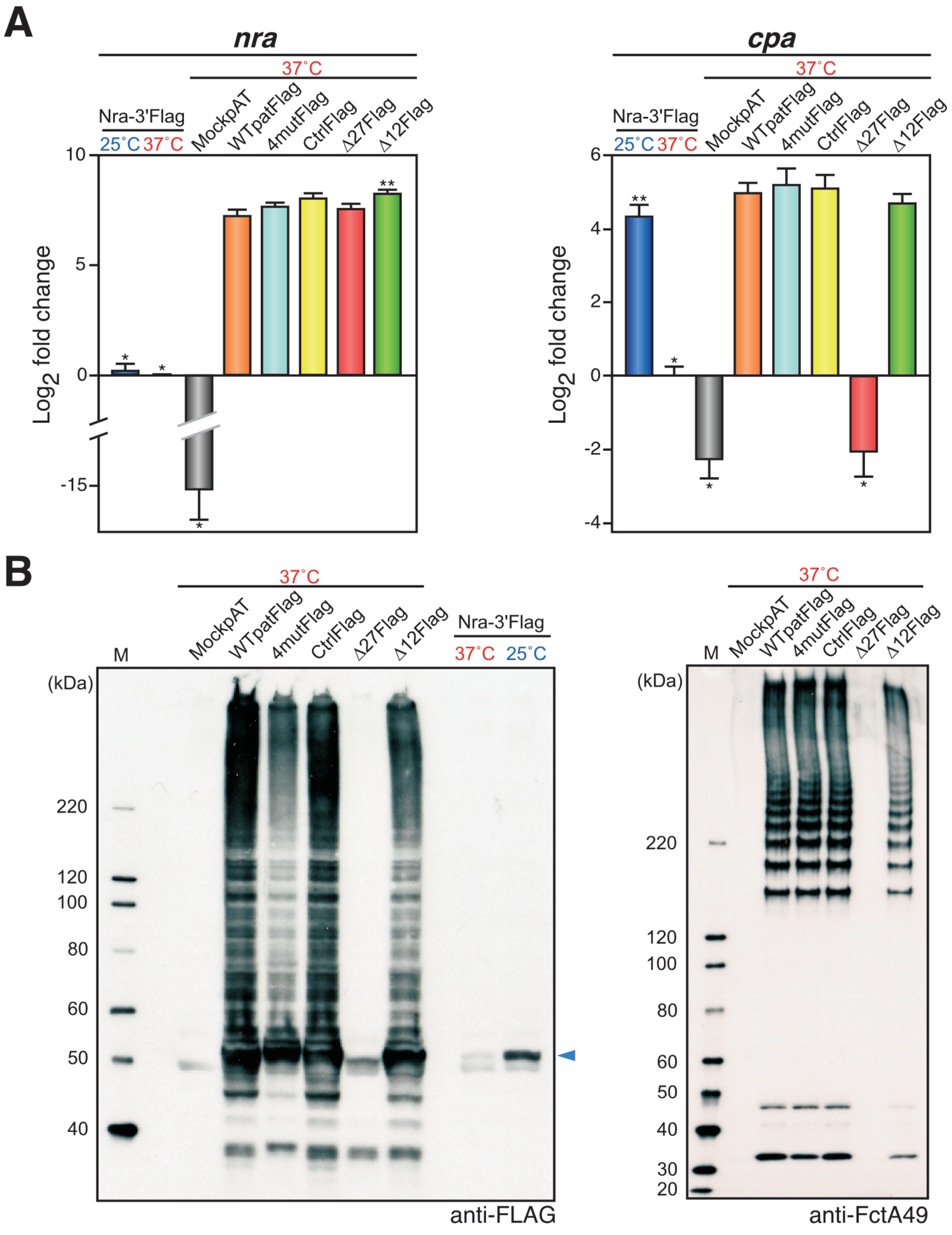

Supplemental Figure 4. The Δ27 mutant Nra is defective in promoting pilus production.
(A) An *nra* in-frame deletion mutant (delNra) was transformed with a pAT-P*gyrA* shuttle vector alone (strain MockpAT), vectors for overexpressing 3'-*flag*-tagged wild-type *nra* (strain WTpatFlag), or a series of 3'-flag-tagged mutant *nra* genes under the *gyrA* promoter to generate 4mutFlag, CtrlFlag, Δ27Flag, and Δ12Flag strains. The introduced mutations are the same as those shown in Figure 7. Note that structural genes were cloned into the vector. Expression of *cpa* and *nra* in the strains, including a strain expressing 3'-*flag* tagged *nra* (Nra-3'Flag) grown to the late-exponential phase (OD_{600} = 0.9) at indicated temperatures, was examined by real time RT-PCR analysis. Results for *gyrA* served as an internal control. Log_2-fold changes in transcription relative to data obtained with Nra-3'Flag grown at 37˚C are shown. Data are presented as the mean ± SD of sextuplet samples and representative of 3 independent experiments. Statistical analysis was performed using Tukey's comparison test with ANOVA. A confidence interval with a *p* value of <0.05 was deemed to indicate significance. Statistical differences between WTpatFlag and other strains are shown. *P <0.01. **P <0.05. (B) Whole cell extracts were prepared from strains grown to the exponential phase (OD_{600} = 0.5) at the indicated temperatures, then Nra was immunodetected with anti-Flag mAb (left panel). Blue arrowhead indicates band corresponding to Flag-tagged Nra. Also, cell wall fractions of strains incubated overnight at 37˚C were immunoblotted with antiserum against FctA49 (right panel). Molecular mass standard sizes are indicated on the left. M, protein size marker.

Supplemental Figure 5.

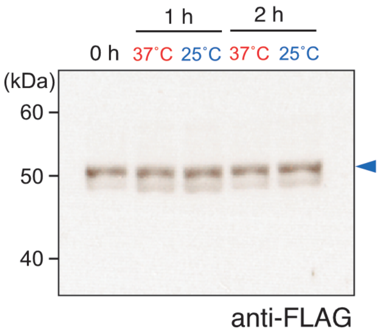

Supplemental Figure 5. Temporal level of Flag-tagged Nra in absence of transcription and translation. Nra-3'Flag cells were grown at 25˚C until an OD_{600} of 0.5. Then, a rifampicin (1 mg/ml) and tetracycline (20 µg/ml) mixture was added, followed by incubation for an additional 2 h at 37˚C or 25˚C. Cells were harvested each hour and whole cell extracts were immunoreacted with anti- Flag mAb. Blue arrowhead indicates band corresponding to Flag-tagged Nra. Molecular mass standard sizes are indicated on the left.

Supplemental Figure 6.

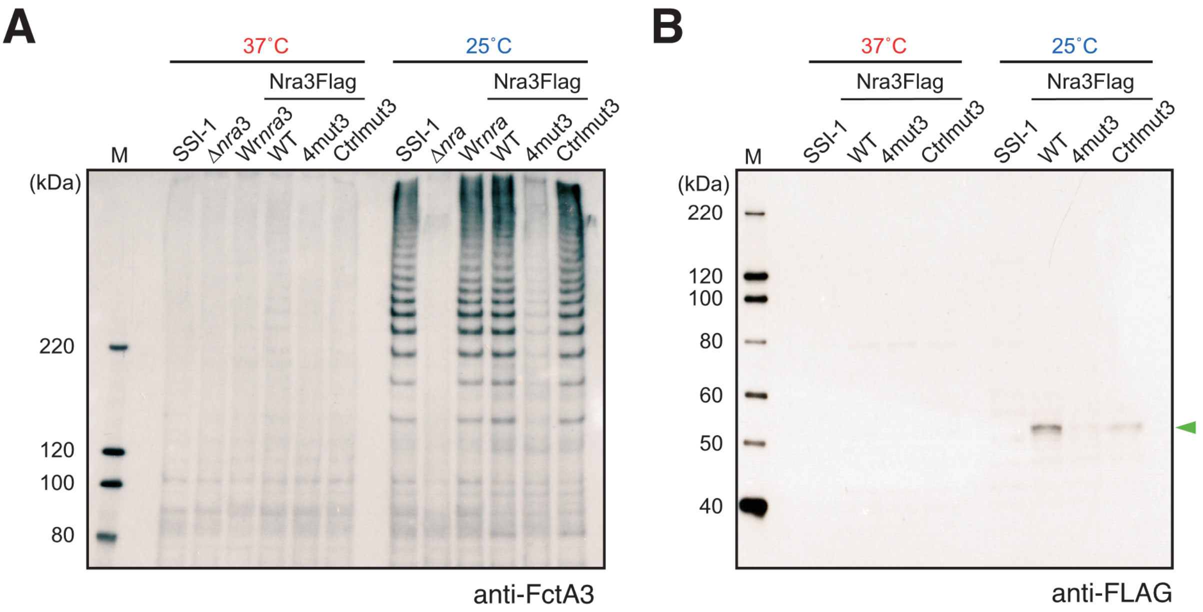

Supplemental Figure 6. Predicted stem-loop structure within coding region of *nra*3 mRNA promotes *nra*3 translation and subsequent pilus production in a serotype M3 strain

(A) An *nra*3 in-frame deletion mutant (Δ*nra*3), a revertant strain possessing wild-type *nra*3 (Wr*nra*3), and a mutant strain chromosomally *flag*-tagged at the 3' end of *nra*3 (Nra3Flag) in the background of the serotype M3 strain SSI-1 were used. Using the same method described for M49 strain 591, chromosomal silent mutations were introduced into either 4 bases of 2 codons encoding D9 and S10 or 4 bases of 2 codons encoding D23 and S25 to generate the 4mut3 and Ctrlmut3 strains, respectively, in the background of Nra3Flag (WT). Cell wall fractions of those strains grown overnight at 37˚C or 25˚C were immunoblotted with antiserum against FctA3. Molecular mass standard sizes are indicated on the left. M, protein size marker. (B) Whole cell extracts were prepared from strains SSI-1, Nra3Flag (WT), 4mut3, and Ctrlmut3 grown at 37˚C or 25˚C to the exponential phase (OD_{600} = 0.5), then immunoblotted using anti-Flag mAb. Green arrowhead indicates band corresponding to Flag-tagged Nra. Molecular mass standard sizes are indicated on the left. M, protein size marker.

Supplemental Table 1. *S. pyogenes* strains used for examination of thermosensitive pilus expression.

emm type	FCT type	Strain	Origin/	Disease	Pilus detection at 37˚C (AS)*	
M1	2	95A-90	Skin	NA	+	anti-FctA1 antiserum
M1	2	TW3362	Throat	Glomerulonephritis	+	anti-FctA1 antiserum
M1	2	SSI-9	NA	TSLS	+	anti-FctA1 antiserum
M1	2	SSI-25	NA	TSLS	+	anti-FctA1 antiserum
M1	2	SF370	Skin	NA	+	anti-FctA1 antiserum
M1	2	TW3348	Tonsil Pus	Tonsillitis	+	anti-FctA1 antiserum
M1	2	SSI-124	NA	TSLS	+	anti-FctA1 antiserum
M1	2	NIH17	NA	TSLS	+	anti-FctA1 antiserum
M2	6	96A-27	Throat	Pharyngitis	+	T2 monovalent
M2	6	96A-51	Throat	Pharyngitis	+	T2 monovalent
M2	6	97A-12	Throat	Pharyngitis	+	T2 monovalent
M2	6	95A-170	Throat	Pharyngitis	+	T2 monovalent
M2	6	95A-179	Throat	Pharyngitis	+	T2 monovalent
M2	6	613	NA	NA	+	Polyvalent U
M2	6	723	NA	NA	+	Polyvalent U
M3	3	TW3358	Throat	Pharyngitis	-	anti-FctA3 antiserum
M3	3	TW3384	Skin	Skin abscess	-	anti-FctA3 antiserum
M3	3	95A-158	Throat	Pharyngitis	-	anti-FctA3 antiserum
M3	3	NIH12	NA	TSLS	-	anti-FctA3 antiserum
M3	3	NIH1	NA	TSLS	-	anti-FctA3 antiserum
M3	3	NIH16	NA	TSLS	-	anti-FctA3 antiserum
M3	3	SSI-7	NA	TSLS	-	anti-FctA3 antiserum
M4	5	96A-104	NA	NA	+	T4 monovalent
M4	5	514	NA	NA	+	Polyvalent U
M4	5	615	NA	NA	+	Polyvalent U
M5	3	574	NA	NA	-	Polyvalent W
M5	3	585	NA	NA	-	Polyvalent W
M5	3	791	NA	NA	-	Polyvalent W
M5	3	792	NA	NA	-	Polyvalent W
M5	3	793	NA	NA	-	Polyvalent W
M5	3	794	NA	NA	-	Polyvalent W
M6	1	SE1387	Throat	Pharyngitis	+	anti-T6 antiserum
M6	1	TW3558	Tonsil Pus	Tonsillitis	+	anti-T6 antiserum
M6	1	SE1303	Throat	Pharyngitis	+	anti-T6 antiserum
M6	1	97A-85	Throat	Pharyngitis	+	anti-T6 antiserum
M6	1	JRS4	NA	NA	+	anti-T6 antiserum
M6	1	576	NA	NA	+	Polyvalent U
M6	1	616	NA	NA	+	Polyvalent U
M8	5	617	NA	NA	+	Polyvalent X
M9	7	541	NA	NA	+	Polyvalent Y
M9	7	542	NA	NA	+	Polyvalent Y
M9	7	543	NA	NA	+	Polyvalent Y
M11	4	544	NA	NA	+	Polyvalent W
M11	4	545	NA	NA	+	Polyvalent W
M11	4	546	NA	NA	+	Polyvalent W
M11	4	619	NA	NA	+	Polyvalent W
M12	4	TW3337	Tonsil Pus	Tonsillitis	+	T12 monovalent
M12	4	94A-1	Throat	Pharyngitis	+	T12 monovalent
M12	4	94A-221	Throat	Pharyngitis	+	T12 monovalent
M12	4	SE1102	Throat	Pharyngitis	+	T12 monovalent
M12	4	SE1416	Throat	Pharyngitis	+	T12 monovalent
M12	4	NIH30	NA	TSLS	+	T12 monovalent
M12	4	620	NA	NA	+	Polyvalent W
M12	4	722	NA	NA	+	Polyvalent W

Supplemental Table 1. continued.

emm type	FCT type	Strain	Origin/	Disease	Pilus detection at 37˚C (AS)*	
M18	3	#43-2	NA	NA	-	anti-FctA18 antiserum
M18	3	TW3363	Throat	Pneumonia	-	anti-FctA18 antiserum
M18	3	TW3537	Throat	Pharyngitis	-	anti-FctA18 antiserum
M18	3	443	NA	NA	-	Polyvalent Y
M18	3	578	NA	NA	-	Polyvalent Y
M18	3	623	NA	NA	-	Polyvalent Y
M23	1	861	NA	NA	+	Polyvalent Y
M23	1	862	NA	NA	+	Polyvalent Y
M23	1	929	NA	NA	+	Polyvalent Y
M25	5	626	NA	NA	+	Polyvalent X
M25	5	863	NA	NA	+	Polyvalent X
M25	5	864	NA	NA	+	Polyvalent X
M28	4	96A-102	Throat	Pharyngitis	+	T28 monovalent
M28	4	95A-81	Skin	Skin infection	+	T28 monovalent
M28	4	TW3415	Skin pus	Impetigo	+	T28 monovalent
M28	4	SE1410	Throat	Pharyngitis	+	T28 monovalent
M28	4	NIH35	NA	TSLS	+	T28 monovalent
M28	4	869	NA	NA	+	Polyvalent U
M28	4	931	NA	NA	+	Polyvalent U
M49	3	591	Skin	Skin infection	-	anti-FctA49 antiserum
M49	3	TW3450	Skin Pus	Tick borne infection	-	anti-FctA49 antiserum
M49	3	CS101	Skin	Impetigo	-	anti-FctA49 antiserum
M49	3	NZ131	NA	Glomerulonephritis	-	anti-FctA49 antiserum
M66	4	927	NA	NA	+	Polyvalent W
M67	3	671	NA	NA	-	Polyvalent T
M71	3	673	NA	NA	-	Polyvalent T

*Immunoblot analysis was performed with the indicated antiserum (AS). Pili were detected in cell wall fractions of all tested strains cultured at 25˚C, except for the M18 strains. Polyvalent and monovalent T typing serum samples were purchased from Denka Seiken. NA, not available. TSLS, Toxic shock-like syndrome.

Supplemental Table 2. Primers used in this study

Primer	Sequence (5'-3')	Purpose
M1-0128F	GCGGATCCGAGACTGTTGTAAACGGAGCCAA	Cloning of FctA1 to pQE30
M1-0128R	GCGTCGACTGTTGGCACTTCAAAGTCTT	Cloning of FctA1 to pQE30
rM3fctAF	GCGGATCCGAGACGGCAGGAGTGTCCGAA	Cloning of FctA3 to pQE30
rM3fctAR	GCCTGCAGTGGAACTTGAGTGTCACGCTT	Cloning of FctA3 to pQE30
rM18fctAF	GCGGATCCGAGACAGCAGGAGTGATTGAT	Cloning of FctA18 to pQE30
rM18fctAR	GCCTGCAGAGTTGGAACTTGAGTGTCACG	Cloning of FctA18 to pQE30
NrakoF1	GCGGATCCTTAAGCTTTATCATTGATAATG	Construction of pSET4-NraKO
NrakoR1	TTAGGAGAGAGCTATTACATGTAAATAACTGACTTTCGTTTA	Construction of pSET4-NraKO
NrakoF2	TAAACGAAAGTCAGTTATTTACATGTAATAGCTCTCTCCTAA	Construction of pSET4-NraKO
NrakoR2	GAGCATGCCAGTATAGTACCAAATAGCATT	Construction of pSET4-NraKO
NraKOcheckF	GCGATGGTACGAAAGGCAGAGTT	Confirmation of *nra* deletion
NraKOcheckR	GCCTAACTGCTGGTCATTAATA	Confirmation of *nra* deletion
NraF/Not	GAGCGGCCGCGCCTTATGTCAAAAAGAAAAAGGA	Construction of pFlag-Nra
NraR/Kpn	GCGGTACCGATTGTAATTGCTCGTTTAGAAAATC	Construction of pFlag-Nra
Nra-flag-F1	GCGGATCCGTTGTAACTGTCCGTCTTCTAT	pSET4-5'Flag-Nra construction
Nra-flag-R1	GTCATGGTCTTTGTAGTCCATGTAATAGCTCTCTCCTAAACT	pSET4-5'Flag-Nra construction
Nra-flag-F2	AGTTTAGGAGAGAGCTATTACATGGACTACAAAGACCATGAC	pSET4-5'Flag-Nra construction
Nra-flag-R2	TAAACGAAAGTCAGTTATTTACAGATCCTCTTCTGAGATGAG	pSET4-5'Flag-Nra construction
Nra-flag-F3	CTCATCTCAGAAGAGGATCTGTAAATAACTGACTTTCGTTTA	pSET4-5'Flag-Nra construction
Nra-flag-R3	GCCTGCAGCAAAGTCATTAGGGCTGCTTCA	pSET4-5'Flag-Nra construction
CflagNraF1	GCCGGATCCGCCAGCCTACAATGGACTCCACA	pSET4-3'Flag-Nra construction
CflagNraR1	CTTGTCATCGTCATCCTTGTAATCGATGTCATGATCTTTATAATCACCGTCATGGTCTTTGTAGTCTTGTAATTGCTCGTTTAGAAA	pSET4-3'Flag-Nra construction
CflagNraF2	GACTACAAAGACCATGACGGTGATTATAAAGATCATGACATCGATTACAAGGATGACGATGACAAGTAAATAACTGACTTTCGTTTA	pSET4-3'Flag-Nra construction
CflagNraR2	GCCCTGCAGGCGTTTTTCATAGCGAGCAATCT	pSET4-3'Flag-Nra construction
CflagNraCheckF	GACCATGACGGTGATTATAAAGA	Confirmation of Nra-3'Flag
CflagNraCheckR	GACGCTTGTAAGGTTCATCTCCA	Confirmation of Nra-3'Flag
nrapmspF1	GCGGATCCAGTCTTATAACTATACTGACA	pMSP-(5'Flag-)Nra construction
nrapmspR1	AGAAGCTAAGAATATTGATATGAAATCATTGTATCTAACAAA	pMSP-(5'Flag-)Nra construction
nrapmspF2	TTTGTTAGATACAATGATTTCATATCAATATTCTTAGCTTCT	pMSP-(5'Flag-)Nra construction
nrapmspR2	GCCTGCAGGTAGAAGCACATACAACTGAATCAGGT	pMSP-(5'Flag-)Nra construction
OperonNraF	GCGGATCCCCAGTAACTGAGAAATGTAGAAGCA	Construction of pAT-Op-Nra
OperonR	GCCTGCAGGGTACAAAAACCTATCAGCATAGAA	Construction of pAT-Op-Nra
pATnracpainvF	GCCGTCGACCTGTTCAAGATATGTTTCTACTA	Construction of pAT-Op
pATnracpainvR	GCCGTCGACCAATTACAATAAATAACTGACTTTC	Construction of pAT-Op
RTnra5P$^{\#}$	GGTTCCTCTAGCCAG	5'-RACE for *nra*
S2nra	GCGTCGACTGTTGGCACTTCAAAGTCTT	5'-RACE for *nra*
S1nra	GCGGATCCGAGACGGCAGGAGTGTCCGAA	5'-RACE for *nra*
A1nra	GCCTGCAGTGGAACTTGAGTGTCACGCTT	5'-RACE for *nra*
A2nra	GCGGATCCGAGACAGCAGGAGTGATTGAT	5'-RACE for *nra*

The primer is 5'-phosphorylated.

Supplemental Table 2. continued.

Primer	Sequence (5'-3')	Purpose
NrarealF	TTAAGAATGATCTGGTCAAAGGTGTT	Real-time RT-PCR
NrarealR	GAAGCAATAGAGTAGTCAGGGTTATCAA	Real-time RT-PCR
CparealF	AGCGTTCGGAGCTGAAGAAC	Real-time RT-PCR
CparealR	GCCATACCACGGATAATCTTGAA	Real-time RT-PCR
GyrAF	CGACTTGTCTGAACGCCAAA	Real-time RT-PCR
GyrAR	TTATCACGTTCCAAACCAGTCAA	Real-time RT-PCR
NraDelta27F1	GCCGGATCCGCAGTATGCTTGATAATCCTTAC	pSET4-Δ27 construction<br>pSET4-Δ12 construction<br>pSET4-4mut construction<br>pSET4-Ctrl construction
NraDelta27R1	CTTTTATCTCTAATAGACTGTTCCTTTTTCTTTTTGACATAA<br>GG	pSET4-Δ27 construction
NraDelta27F2	CCTTATGTCAAAAAGAAAAAGGAACAGTCTATTAGAGATA<br>AAAG	pSET4-Δ27 construction
NraDelta27R2	GCCCTGCAGGAGATCTCCCTGTGAGAACGTTA	pSET4-Δ27 construction<br>pSET4-Δ12 construction<br>pSET4-4mut construction<br>pSET4-Ctrl construction
NraDelta12R1	CTGTTCAAGATATGTTTCTACCTTTTTCTTTTTGACATAAGG	pSET4-Δ12 construction
NraDelta12F2	CCTTATGTCAAAAAGAAAAAGGTAGAAACATATCTTGAAC<br>AG	pSET4-Δ12 construction
NraPoint4mutR1	GTTTCTACTAAGAATGAGTCCTTTTTCT	pSET4-4mut construction
NraPoint4mutF2	AGAAAAAGGACTCATTCTTAGTAGAAAC	pSET4-4mut construction
4mutCtrlR1	AGACTAATTCTGATTTGTCTCTAATAG	pSET4-Ctrl construction
4mutCtrlF2	CTATTAGAGACAAATCAGAATTAGTCT	pSET4-Ctrl construction
Delta27checkF	GATCTAACACTATCTGACTTGGA	Confirmation of Δ27<br>Confirmation of Δ12<br>Confirmation of 4mut<br>Confirmation of Ctrl
NraMutCheckR	TCAAGATATGTTTCTACTAAGAA	Confirmation of Δ27<br>Confirmation of Δ12
NraPointCheckR	GATATGTTTCTACTAAGAAAC	Confirmation of 4mut
4mutCtrlcheckR	TAATAGTAAGACTAATTCACT	Confirmation of Ctrl
NraseqR3	GTCGATAAGCTGAGGAGCTTG	Confirmation of Δ27<br>Confirmation of Δ12<br>Confirmation of 4mut<br>Confirmation of Ctrl
NraseqR3	GTTCTTCAGCTCCGAACGCTC	Confirmation of Δ27<br>Confirmation of Δ12<br>Confirmation of 4mut<br>Confirmation of Ctrl
nrainvitroF	CTTAATACGACTCACTATAGGGATATCAATATTCTTAG<br>CTTCTAA	*In vitro* transcription
nrainvitroR	GAAATGTAGAAGCACATACAA	*In vitro* transcription
nrainvitroFtac	GAGCTGTTGACAATTAATCATCGGCTCGTATAATGTG<br>TGGAATATCAATATTCTTAGCTTCTAA	*In vitro* transcription

Supplemental Table 2. continued.

Primer	Sequence (5'-3')	Purpose
Nra349F	GGCGGATCCATGCCTTATGTCAAAAAGAAAAAGG	Cloning with pAT18-P*gyrA*
Nra349R2	GCCCTGCAGGAATCAGGTTAAATGAATAACAGA	Cloning with pAT18-P*gyrA*
NraM3_4mutF1	GCCGGATCCGCAGTATGCTTGATACTCCTTAC	Construction of 4mut3
NraM3_4mutR2	GCCCTGCAGGAGATCTCCTTGTGAGAACGTTA	Construction of 4mut3
CtrlmutCheckR	TAACAGTAAGACTAATTCACT	Confirmation of Ctrlmut3
